# Biomimetic Scaffold‐Based 3D Models for Decoding Cancer Biology and Advancing Therapy

**DOI:** 10.1002/advs.77715

**Published:** 2026-09-16

**Authors:** Haitao Zhao, Yuechen Han, Jie Li, Peng Luo, Lin Qi, Zhiwei Xia, Xiaosong Li, Quan Cheng, Chenshen Huang, Wantao Wu, Hao Zhang

**Affiliations:** ^1^ Department of Neurosurgery The Second Affiliated Hospital Chongqing Medical University Chongqing People's Republic of China; ^2^ Department of Neurology The First Affiliated Hospital Chongqing Medical University Chongqing People's Republic of China; ^3^ Department of Clinical Laboratory Children's Hospital of Chongqing Medical University Chongqing People's Republic of China; ^4^ Department of Oncology Zhujiang Hospital Southern Medical University Guangzhou People's Republic of China; ^5^ Department of Orthopedics The Second Xiangya Hospital Central South University Changsha People's Republic of China; ^6^ Hunan Key Laboratory of Tumor Models and Individualized Medicine The Second Xiangya Hospital Changsha People's Republic of China; ^7^ Department of Neurology Hunan Aerospace Hospital Hunan Normal University Changsha People's Republic of China; ^8^ Department of Neurology Hunan Aerospace Hospital Changsha Medical University Changsha People's Republic of China; ^9^ Clinical Molecular Medicine Testing Center Key Laboratory of Clinical Laboratory Diagnostics (Chinese Ministry of Education) The First Affiliated Hospital of Chongqing Medical University Chongqing People's Republic of China; ^10^ Western Institute of Digital‐Intelligent Medicine Chongqing People's Republic of China; ^11^ Department of Neurosurgery Xiangya Hospital Central South University Changsha People's Republic of China; ^12^ Department of Neurosurgery Xiangya School of Medicine Central South University Changsha People's Republic of China; ^13^ Department of Neurosurgery Xiangya Hospital, National Regional Center for Neurological Diseases Central South University Nanchang Jiangxi People's Republic of China; ^14^ National Clinical Research Center for Geriatric Diseases (Xiangya Hospital) Central South University Changsha People's Republic of China; ^15^ Department of Gastrointestinal Surgery Fuzhou University Affiliated Provincial Hospital School of Medicine, Fuzhou University Fuzhou People's Republic of China; ^16^ Department of Thyroid and Breast Surgery The Second Affiliated Hospital Chongqing Medical University Chongqing People's Republic of China

**Keywords:** 3D tumor models, biomimetic biomaterials, cancer therapeutics, scaffold‐based culture, tumor microenvironment

## Abstract

While conventional two‐dimensional (2D) cultures and animal models remain essential tools, they frequently fail to recapitulate the three‐dimensional (3D) architecture, biomechanical cues, and spatial complexity of human tumors, thereby limiting their translational relevance. To address these limitations, scaffold‐based 3D culture systems have emerged as powerful platforms that leverage engineered biomaterials to mimic key physical and biochemical properties of the native tumor microenvironment (TME). This review systematically examines the latest advances in biomimetic scaffold‐based 3D tumor models. We first outline the principal biomaterials used in scaffold fabrication, including natural and synthetic polymers, hybrid composites, and decellularized extracellular matrix (dECM). We then discuss scaffold design strategies to replicate key hallmarks of cancer, including matrix stiffness, hypoxia, metabolic gradients, viscoelasticity, cell adhesion, proteolytic remodeling, and multicellular crosstalk. Furthermore, we highlight the application of these models in drug screening, personalized medicine, radiotherapy testing, and the study of metastasis and recurrence. Finally, we address persistent challenges in standardization, scalability, and clinical translation, while offering perspectives on future directions, including 4D bioprinting, smart responsive materials, multi‐omics integration, and the development of “clinical trial in a dish” platforms.

## Graphical abstract

A timeline of the research history of 3D biomimetic scaffold materials and platforms. With advances in biomimetic materials and 3D scaffold technologies, their functional scope has expanded from mimicking basic physical and biochemical properties to increasingly replicating complex pathophysiological processes. Contemporary 3D biomimetic scaffolds enable the construction of sophisticated ECM architectures that more closely recapitulate the native in vivo microenvironment.

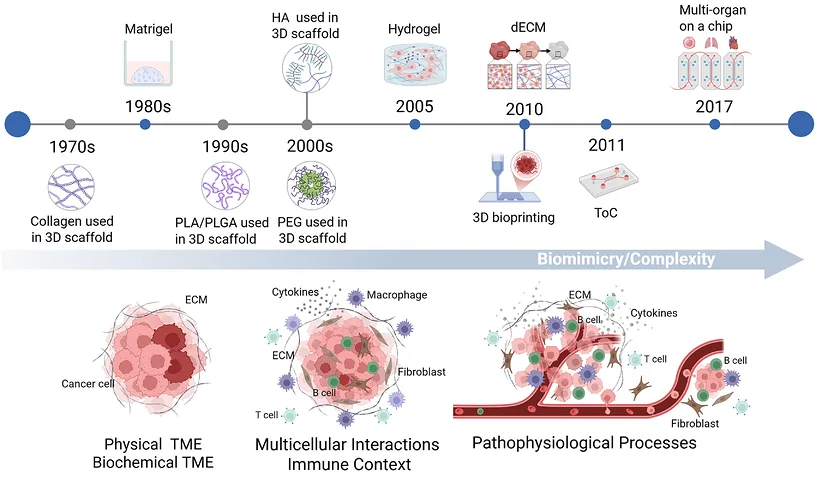

**Abbreviations**
2D: Two‐dimensional
3D: Three‐dimensional
α‐SMA: α‐smooth muscle actin
ACT: Adoptive cell transfer
ADAM: A disintegrin and metalloproteinas
BC: Breast cancer
CAA: Cancer‐associated adipocyte
CAF: Cancer‐associated fibroblast
CSC: Cancer stem cell
CTC: Circulating tumor cell
DAMP: Damage‐associated molecular pattern
dECM: Decellularized extracellular matrix
DHCM: Decellularized hepatocellular carcinoma matrix
DLM: Decellularized liver matrix
EC: Endothelial cell
ECM: Extracellular matrix
EGF: Epidermal growth factor
EMT: Epithelial‐mesenchymal transition
ERα: Estrogen receptor α
FAK: Focal adhesion kinase
GBM: Glioblastoma
GelMA: Gelatin methacryloyl
HA: Hyaluronic acid
HAMA: Methacrylated hyaluronic acid
HL: Hodgkin lymphoma
HUVEC: Human umbilical vein endothelial cell
ICI: Immune checkpoint inhibitor
MMP: Matrix metalloproteinase
MSC: Mesenchymal stem cell
PCL: Poly(ε‐caprolactone)
PD‐1: Programmed cell death protein 1
PDAC: Pancreatic ductal adenocarcinoma
PDMS: Polydimethylsiloxane
PDS: Patient‐derived scaffold
PEG: Poly(ethylene glycol)
PEGDA: Poly(ethylene glycol) diacrylate
PF: Papillary fibroblast
PLA: Polylactic acid
PLGA: Poly(lactic‐co‐glycolic acid)
PSC: Pancreatic stellate cell
RF: Reticular fibroblast
TAM: Tumor‐associated macrophage
TME: Tumor microenvironment
TNBC: Triple‐negative breast cancer
ToC: Tumor‐on‐a‐chip
VASP: Vasodilator‐stimulated phosphoprotein
VE‐cadherin: Vascular endothelial cadherin
VM: Vasculogenic mimicry

## 1 Introduction

Tumors are not merely aggregates of malignant cells but highly complex ecosystems whose initiation, progression, and therapeutic resistance are critically shaped by a dynamic tumor microenvironment (TME). The TME comprises cancer cells, diverse stromal cell populations, a remodeled extracellular matrix (ECM), and interconnected biochemical and biophysical signaling networks. Cancer cells actively recruit and reprogram surrounding host cells while restructuring the vascular network and ECM, thereby establishing a permissive niche that supports proliferation, invasion, metastasis, and resistance to therapy [1].

Historically, cancer research has relied on two‐dimensional (2D) cell culture systems and animal models. However, both approaches exhibit intrinsic limitations in reproducing the complexity and heterogeneity of human TMEs. Although animal models provide an integrated physiological context, interspecies differences often limit their predictive accuracy for human responses, and their use is poorly suited to high‐throughput screening or detailed mechanistic analyses [2]. In contrast, 2D cultures lack the three‐dimensional (3D) architecture and biomechanical properties of native tissues, failing to capture essential cell‐ECM interactions and spatial heterogeneity that drive tumor progression, metastatic dissemination, and treatment resistance [3].

To address these shortcomings, the field has increasingly shifted toward 3D cell culture models. These systems aim to reproduce the biological features of patient tumors, thereby narrowing the gap between simplistic monolayer cultures and complex in vivo models. Among available approaches, biomimetic scaffold‐based 3D culture systems offer distinct advantages. Unlike scaffold‐free models such as spheroids or hanging‐drop cultures, scaffold‐based platforms incorporate synthetic, biomimetic ECMs that are deliberately engineered using principles from biomaterials science and tissue engineering. These matrices enable the in vitro reconstruction of key components of the TME, including cellular heterogeneity, ECM composition, and biophysical signaling. Culturing cancer cells within such engineered scaffolds supports 3D growth, migration, and cell–cell interactions that more closely resemble in vivo conditions. As a result, these systems provide a more accurate representation of the TME's physical and biochemical properties, creating a powerful platform for investigating tumor pathophysiology and therapeutic responses [4, 5].

This review systematically examines recent advances and the emerging potential of 3D biomimetic scaffold models in cancer research. We first outline the current “biomimetic scaffold toolkit,” then discuss how rational scaffold design can be used to recapitulate key hallmarks of cancer in vitro. We further highlight applications of these models in translational cancer research, including drug screening, therapeutic discovery, and personalized medicine. Finally, we address existing challenges and outline future directions for this rapidly evolving field.

## 2 Methods

### 2.1 Information Sources and Search Strategy

This study was conducted as a structured narrative review based on a comprehensive literature search. Literature published from January 1995 to January 2026 was retrieved from the PubMed and Web of Science electronic databases. The strategy combined three concept blocks with Boolean AND: three‐dimensional cancer models (“3D culture,” “three‐dimensional culture,” “tumor model,” “cancer model,”); scaffold‐related biomaterials and engineering platforms (“scaffold,” “hydrogel,” “microfluidic,” “organ‐on‐a‐chip,” “3D bioprinting,” “spheroids”); and oncology terms (“neoplasms,” “cancer,” “tumor,” “carcinoma”). We hand‐searched reference lists of pertinent review articles to locate additional studies.

### 2.2 Eligibility Criteria

Inclusion: (i) Studies investigating scaffold‐based 3D tumor models for cancer research; (ii) Studies reporting the design, characterization, and biological validation of scaffold‐based tumor models; (iii) articles published in English.

Exclusion: (i) Studies focusing exclusively on scaffold‐free 3D models; (ii) organoid models; (iii) Studies based solely on conventional 2D cell culture, animal models, or in silico simulations.

## 3 The Biomimetic Scaffold Toolkit: Engineering the Extracellular Niche

The development of 3D cell culture technologies that recapitulate the TME is a central objective in contemporary cancer research. Existing 3D tumor models can be broadly classified into scaffold‐free multicellular tumor spheroids and scaffold‐based systems. Scaffold‐free multicellular tumor spheroid culture is an approach in which cells self‐assemble into 3D structures without exogenous supporting materials. It relies on spontaneous aggregation under suspension or non‐adherent conditions, driven by cell–cell adhesion and cell‐secreted ECM [4]. By comparison, scaffold‐based systems employ natural or synthetic biomaterials onto which cells are seeded or within which they are encapsulated. These materials provide mechanical support and biochemical cues that promote cell growth, proliferation, migration, and spatial organization while more closely recapitulating selected features of the native ECM. Due to length constraints, this review focuses primarily on scaffold‐based models of 3D tumor cell cultures.

Given the pronounced cellular, structural, and biochemical heterogeneity of the TME, the complexity of human tumors cannot be fully recapitulated by any single 3D platform. Integrating biomaterials with advanced technologies and culture platforms has substantially expanded the capabilities of scaffold‐based systems, enabling the development of diverse models that reproduce distinct physiological and pathological features observed in vivo. Based on the manner in which scaffolds are integrated with tumor cells and culture systems, these models can be broadly categorized into hydrogel‐encapsulated tumor models, scaffold‐integrated microfluidic models, bioprinted scaffold‐based tumor models, and scaffold‐supported spheroid models. Their fundamental principles, major advantages, limitations, and representative applications are summarized in Table 1.

**TABLE 1:** Description, advantages, and limitations of common scaffold‐based 3D cell culture techniques and platforms.

Techniques and platforms	Description	Advantages	Limitations	References
Hydrogel‐encapsulated tumor models	The hydrogel scaffold consists of hydrophilic polymer chains forming a 3D network structure in a water‐rich environment. Based on the origin of the biomaterials, hydrogels can be classified as natural or synthetic.	Hydrophilicity and biocompatibility Gel properties, including pore size and biodegradation rate, can be tailored. Allowing nutrient and oxygen diffusion and waste removal. Allowing soluble factors such as cytokines and growth factors to travel through the tissue‐like gel.	Limited mechanical strength. Lack of long‐term stability and limited control over microstructure.	[6, 7, 8]
Scaffold‐integrated microfluidic models	Cells are cultured within biomaterial scaffolds incorporated into microfluidic chambers and supplied through perfusion channels. The scaffold and microchannel system recreate 3D tumor architecture, restricted mass transport, and spatial gradients of oxygen, nutrients, pH, and cellular activity.	Precise regulation of perfusion and microenvironmental gradients. Improved simulation of cell–cell and cell–ECM interactions. Reproduction of spatial tumor heterogeneity, hypoxia, acidosis, and necrosis. Compatibility with spatial analysis, mechanistic studies, and drug screening. Strong controllability and large data volume.	Technically demanding device fabrication and operation; Complex integration of scaffolds and perfusion systems High cost and limited scalability	[9, 10, 11]
Bioprinted scaffold‐based tumor models	Cells are embedded in an ECM‐mimetic hydrogel scaffold and spatially deposited by inkjet‐based, extrusion‐based, and light‐assisted bioprinting. Scaffold composition, architecture, porosity, and stiffness can be adjusted, and tumor cells may be printed alone or together with stromal, immune, neural, and vascular cells.	Precise spatial organization and controllable biochemical and mechanical properties; Improved cell viability and long‐term culture; Enhanced cell–cell and cell–ECM interactions; Incorporation of multiple cell types and vascular structures;	Strong dependence on bioink formulation and crosslinking; Possible nutrient and oxygen diffusion limitations; Scaffold‐induced alterations in cell behavior;	[12, 13]
Scaffold‐supported spheroid models	Tumor spheroids are generated or embedded within natural, synthetic, or hybrid hydrogel matrices that provide ECM‐like biochemical and mechanical cues.	Recapitulates interactions between tumor cells and the ECM under in vivo‐like conditions. The composition and properties of semisynthetic or synthetic hydrogels can be precisely controlled, thereby improving experimental reproducibility. Hybrid hydrogels combine the biological activity of natural materials with the stability and controllability of synthetic matrices.	The chemical composition of biological matrices varies among batches, resulting in poor experimental reproducibility. Spheroids with heterogeneous sizes are readily formed, complicating drug‐screening and treatment‐response studies. Some synthetic hydrogels are intrinsically bioinert and nonbiodegradable. Material development and purification remain complex and costly.	[14]

Although advanced technological platforms enhance the complexity and controllability of scaffold‐based 3D cultures, the choice of biomaterial influences the physicochemical and biological characteristics of tumor models, including mechanical stiffness, porosity, degradation behavior, ligand availability, and cell‐adhesive properties. These parameters directly regulate tumor cell proliferation, migration, invasion, and therapeutic sensitivity. Therefore, a comprehensive understanding of the unique properties of different biomaterials is essential for rational platform selection and the design of models tailored to specific research objectives (Figure 1). Accordingly, this section reviews the major classes of biomaterials employed in scaffold‐based 3D cultures, with an emphasis on their applications in advanced tumor modeling (Table 2).

**FIGURE 1:**
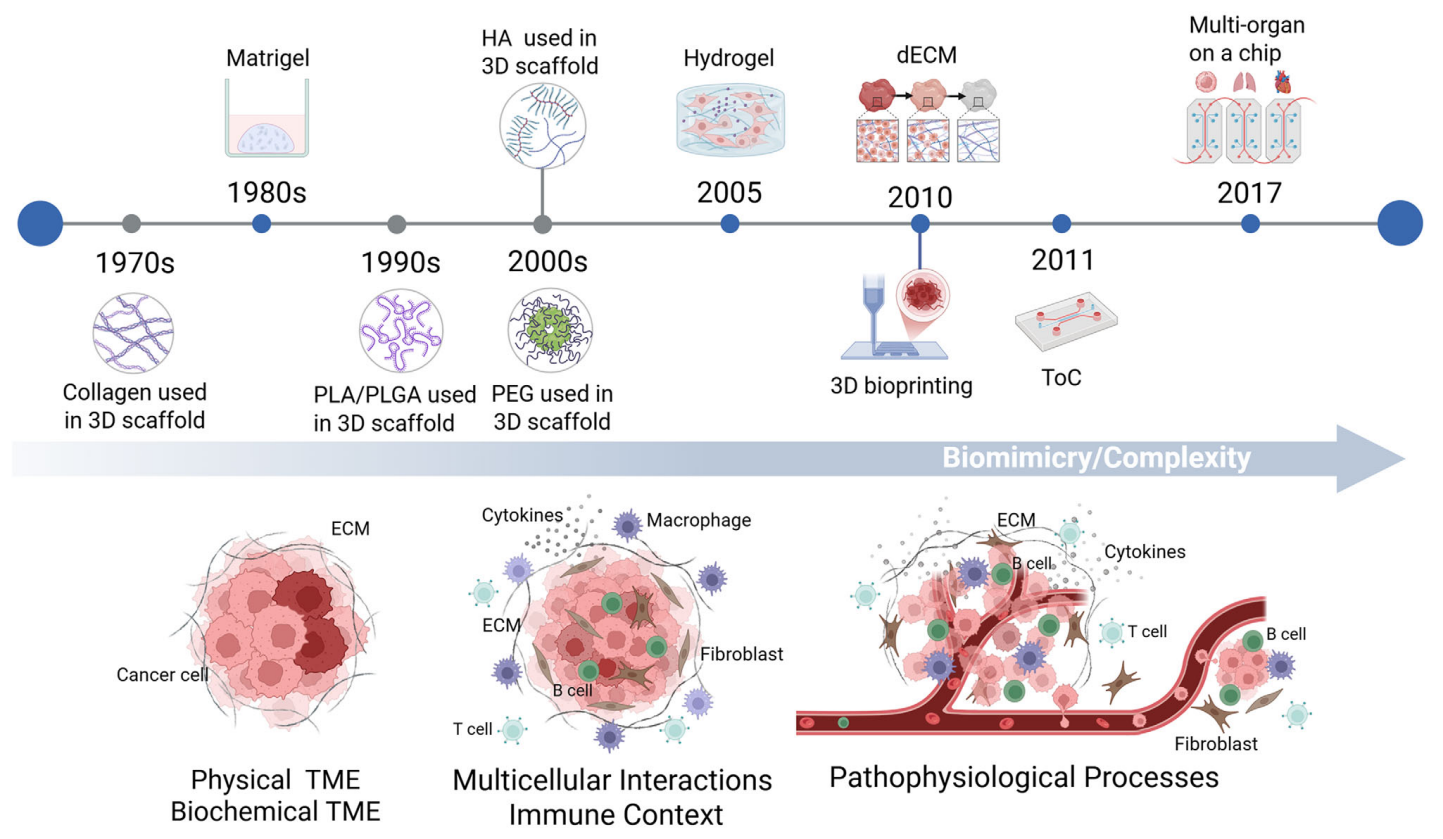
A timeline of research history of 3D biomimetic scaffold materials and platforms. With advances in biomimetic materials and 3D scaffold technologies, their functional scope has expanded from mimicking basic physical and biochemical properties to increasingly replicating complex pathophysiological processes. Contemporary 3D biomimetic scaffolds enable the construction of sophisticated ECM architectures that more closely recapitulate the native in vivo microenvironment. Toc, tumor on‐a‐chip; dECM, decellularized extracellular matrix; HA, hyaluronic acid; PLA, polylactic acid; PLGA, poly(lactic‐co‐glycolic acid); PEG, poly(ethylene glycol).

**TABLE 2:** Summary of biomaterials used in scaffold‐based 3D cultures.

Type	Material	Feature
Natural polymer	Matrigel [21, 34, 40, 58]	A complex mixture secreted by Engelbreth‐Holm‐Swarm mouse sarcoma, rich in growth factors and ECM proteins such as laminin and collagen IV, which mimics the basement membrane and provides a good mimic of in vivo ECM. It exhibits poor stability and undergoes rapid biodegradation, rendering it generally unsuitable for long‐term culture. Furthermore, significant batch‐to‐batch variation substantially limits its broader application.
	Collagen [17, 21, 25, 33, 83]	Collagen is the principal structural protein of the mammalian ECM and provides essential mechanical support to cells. As the most abundant ECM protein, recombinant type I collagen hydrogels are widely employed as in vitro models. It exhibits excellent biocompatibility while eliciting minimal immunogenic responses. Its intrinsic RGD motifs provide native cell‐adhesion sites, thereby facilitating cell attachment, interaction, proliferation, and migration. These properties are critical for maintaining cell viability and for the construction of a physiologically relevant 3D biomimetic scaffold. It has relatively poor mechanical strength, limited long‐term stability, and high production costs. To address these issues, chemical or physical cross‐linking strategies are commonly applied to improve its mechanical performance and durability. In addition, collagen is frequently combined with other polymers, such as alginate or gelatin, to synergistically enhance printability, mechanical robustness, and biological functionality. Collagen can also function as a bioink. Purified collagen solutions, particularly type I collagen, exhibit thermogelling behavior and are therefore widely used in 3D bioprinting. It has potential biosafety concerns remain. Conventional collagen is typically extracted from bovine or porcine tissues and thus carries a theoretical risk of transmitting animal‐derived pathogens.
	Gelatin [84, 85, 86]	Gelatin, a partially hydrolyzed derivative of collagen, exhibits excellent biocompatibility, favorable cell‐adhesion properties, and inherent biodegradability. Compared with native collagen, gelatin induces a lower antigenic response while retaining key RGD motifs that promote cell adhesion, as well as MMP‐sensitive cleavage sites. Together, these features provide effective support for cellular attachment and remodeling. Gelatin is readily available and can be extracted from diverse sources, making it more cost‐effective than other ECM‐derived proteins such as collagen, laminin, and fibronectin. In addition, gelatin offers versatile photocrosslinking strategies, allowing the fabrication of customized hydrogel scaffolds with precisely tunable biomechanical properties.
	Alginate [69, 87, 88, 89]	Alginate is a naturally occurring anionic polysaccharide extracted from brown algae and consists of linear chains of β‐(1–4)‐D‐mannuronic acid and α‐(1,4)‐L‐guluronic acid. It is stable at ambient temperature, nontoxic, and readily forms hydrogels, while avoiding concerns associated with animal‐derived materials. Owing to its good biocompatibility and minimal immunogenicity, alginate is widely used as a hydrogel matrix for constructing 3D cell culture systems that mimic the TME. Cell survival and functional activity are often limited in native alginate‐based 3D cultures. Alginate is biologically inert and lacks intrinsic cell‐binding motifs, resulting in poor cell adhesion and spreading. Thus, it is commonly modified or blended with bioactive components to enhance cell–matrix interactions such as gelatin or RGD peptides.
	Hyaluronic acid [37, 79, 90]	HA is a naturally occurring, anionic linear glycosaminoglycan and a major component of the ECM in brain tissue, where it is abundantly localized at cell surfaces. In addition to its structural role within the tumor ECM, HA functions as a bioactive molecule that can promote tumor progression by regulating cell‐signaling pathways. HA contributes to tissue hydration and mechanical buffering and is biodegradable, non‐immunogenic, and non‐inflammatory. Owing to its excellent biocompatibility, distinctive biological functions, and ease of chemical modification, HA is an attractive matrix material for the construction of 3D tumor models. Conventional HA hydrogels based on single cross‐linked networks often exhibit limited structural integrity and suboptimal mechanical properties. To overcome these limitations, a range of HA derivatives and cross‐linking strategies have been developed. Among them, photopolymerizable HA derivatives are particularly favored, as they provide a balanced combination of mechanical strength, structural stability, and biosafety.
	Silk fibroin [19, 45]	Silk fibroin is a structural fibrous protein derived from silk that provides exceptional and tunable long‐term mechanical support. It is biocompatible and non‐cytotoxic, with intrinsic self‐assembly capabilities and a robust molecular structure. When combined with gelatin and nano‐hydroxyapatite, silk fibroin‐based composites exhibit significantly enhanced mechanical strength.
	Chitosan [34, 88, 91]	Chitosan is a naturally occurring polysaccharide obtained through the N‐deacetylation of chitin. It shares structural similarities with glycosaminoglycans and is most commonly derived from crustacean shells. Chemically, chitosan is composed of 1,4‐linked 2‐acetamido‐2‐deoxy‐β‐D‐glucopyranosyl (N‐acetyl‐β‐D‐glucosaminyl) units. Chitosan is widely used in biomedical applications due to its favorable physicochemical and biological properties, including excellent biocompatibility, biodegradability, and hydrophilicity, which collectively make it a highly suitable biomaterial.
Synthetic Polymer	Poly(lactic‐co‐glycolic acid) [16, 52, 92]	Degradation of PLGA generates acidic by‐products that can influence cellular behavior and therefore require careful consideration in biomedical applications. PLGA is widely available, cost‐effective, and well‐suited for large‐scale 3D bioprinting and the fabrication of certain tissue constructs. Its physicochemical properties can be readily tailored to meet the specific mechanical and structural requirements of different culture systems. To enhance bioactivity, PLGA scaffolds are frequently coated with ECM proteins to provide cell‐recognition and adhesion cues.
	Polyethylene glycol [22, 55, 93]	PEG is a widely used biomaterial characterized by excellent biocompatibility, high hydrophilicity, low nonspecific protein and cell adsorption, and minimal immunogenicity. It is intrinsically bioinert and is therefore frequently modified or combined with bioactive components, such as alginate or RGD motifs, to enhance cell adhesion and biological interactions.
	Poly(ε‐caprolactone) [53, 94, 95, 96]	PCL is one of the most widely used biopolymers due to its favorable mechanical properties and relatively slow biodegradation rate. It has been shown to be biocompatible and free of cytotoxic components, making PCL‐based scaffolds well suited as solid supports for 3D cancer cell culture. It is frequently employed in electrospinning applications because of its suitable viscoelastic properties, tunable melting temperature, and excellent biocompatibility. These characteristics enable the fabrication of fibrous scaffolds that are highly compatible with cell culture systems. As an FDA‐approved biocompatible polymer, PCL can be readily functionalized with bioactive surface coatings. For example, polydopamine‐modified PCL scaffolds have been reported to enhance osteogenic and angiogenic differentiation of human mesenchymal stem cells (MSC), underscoring their potential utility in bone tissue engineering.
	Polylactic acid [97]	This thermoplastic polymer exhibits the highest mechanical strength among biodegradable polymers but is inherently brittle. Its toughness can be effectively enhanced by blending with PCL or PU. It lacks intrinsic bioactivity. Incorporation of bioactive components such as PEG, HA, or chitosan can improve its biological performance. During degradation, the polymer releases acidic by‐products that may generate a localized acidic microenvironment. Such conditions can negatively impact cell viability and cellular function.
	Polyurethane [31]	PUs are highly promising biomaterials due to their unique segmented molecular architecture, wide spectrum of tunable mechanical and physical properties, versatility ranging from stable to biodegradable formulations, and inherent biocompatibility.

### 3.1 Natural Polymer‐Based Scaffolds

Natural polymers derived from biological sources are foundational materials for biomimetic 3D culture systems [15]. They typically require minimal processing and exhibit excellent biocompatibility, biodegradability, and intrinsic bioactivity, all of which support essential cellular functions [16]. These materials are broadly categorized into protein‐based and polysaccharide‐based polymers, which play complementary structural and functional roles within the ECM.

Protein‐based polymers, including collagen [17], fibrin [18], silk fibroin [19], and Matrigel [20], possess strong cell‐instructive properties. They contain specific amino acid sequences [21, 22, 23], most notably the Arg‐Gly‐Asp (RGD) motif, which mediates integrin‐dependent signaling involved in cell adhesion, proliferation, and differentiation [24, 25, 26]. In addition, their susceptibility to degradation by cell‐secreted enzymes such as matrix metalloproteinases (MMPs) enables dynamic, cell‐driven remodeling of the scaffold [27], a process that is essential for cancer invasion and angiogenesis [28, 29, 30]. Despite these advantages, protein‐based hydrogels generally exhibit limited mechanical strength, forming soft matrices that often fail to replicate the stiffness of certain native tissues or advanced TMEs [27, 31].

In contrast, polysaccharide‐based polymers, such as hyaluronic acid (HA) [32], alginate [33], and chitosan [34], provide highly tunable structural support [24]. These materials form hydrated 3D networks that define physical space for cell growth, and their stiffness, porosity, and elasticity can be precisely adjusted through polymer concentration or crosslinking strategies, including ionic and covalent bonding [35]. However, most polysaccharides lack inherent cell‐adhesive motifs [36]. To facilitate cell attachment, they often require functionalization with specific peptides [37, 38].

Natural polymers can be processed into diverse scaffold formats, including hydrogels and fibrous constructs, depending on experimental requirements [25, 34, 39]. The choice of material and the form of scaffold should be guided by the biological question under investigation. For instance, collagen I is widely used to model cancer cell invasion because its fibrous architecture necessitates protease‐mediated degradation during cell migration [7]. Matrigel, which is enriched in laminin, is commonly used to study cancer stem cell (CSC) niches and angiogenesis due to its potent pro‐growth and pro‐differentiation signals [40]. In contrast, high‐throughput drug screening often favors synthetic polymers due to their defined composition, mechanical stability, and superior reproducibility during prolonged culture [41, 42].

Furthermore, some natural polymers can be processed or modified to achieve improved biological properties. Gelatin, a partially hydrolyzed derivative of collagen, elicits reduced antigenicity while retaining RGD motifs and MMP‐sensitive degradation sites. Gelatin methacryloyl (GelMA), a photocrosslinkable gelatin derivative, preserves these biological features [43]. This system undergoes photocuring through an irreversible mechanism, featuring controllable mechanical properties, viscoelasticity, excellent biocompatibility, and numerous cell adhesion sites that promote cell growth and endothelial angiogenesis. Consequently, GelMA is widely used in 3D tumor models [44].

The optimal strategy for constructing highly complex TMEs that mimic the in vivo environment often involves integrating protein‐based polymers with polysaccharide‐based polymers, creating protein–polysaccharide composite scaffolds [45, 46, 47]. This approach not only provides essential biological signals but also achieves desirable structural and mechanical properties.

Thus, natural polymers provide a biologically rich foundation for TME modeling, yet their inherent variability and mechanical limitations necessitate careful selection and often hybridization with other materials. Future efforts should focus on standardizing extraction and functionalization processes to enhance reproducibility while preserving bioactivity. Ultimately, integrating multi‐material approaches will be key to balancing biological fidelity with structural robustness in complex tumor models.

### 3.2 Synthetic Polymer‐Based Scaffolds

Synthetic polymers include poly(ethylene glycol) (PEG) [48], poly(lactic‐co‐glycolic acid) (PLGA) [49], and poly(ε‐caprolactone) (PCL) [50]. Their synthesis and modification can be precisely controlled to dictate key parameters, including mechanical properties, degradation kinetics, and chemical structure [51, 52]. By modulating polymer concentration, molecular architecture, and crosslinking density, the stiffness and elasticity of the scaffold can be tuned over a wide range, enabling a systematic investigation of the mechanical cues that influence cancer cell behavior.

Despite these advantages, some synthetic polymers present biocompatibility challenges. Degradation of polymers such as polylactic acid (PLA) and PLGA generates acidic by‐products, including lactic acid, which can create localized acidic microenvironments detrimental to cell viability and function [16]. In contrast, PCL degrades slowly, typically over more than one year, and produces neutral by‐products, making it suitable for long‐term implantation and culture studies [53]. Additionally, many synthetic polymers, particularly PEG, are intrinsically bioinert and lack cell‐interacting signals [54]. To address this limitation, biofunctionalization strategies are widely employed. Conjugation of RGD peptides introduces cell adhesion sites [26, 55, 56]; incorporation of MMP‐cleavable sequences enables cell‐mediated degradation and invasion [48]; and controlled delivery of growth factors guides differentiation and angiogenesis [57]. Driven by these challenges, researchers have developed novel biomaterials with significantly enhanced biocompatibility. Polyurethanes (PUs), for example, exhibit segmented block structures that confer a broad range of tunable mechanical properties, variable degradability, and demonstrated cytocompatibility, making them promising candidates for tissue engineering applications [31].

Overall, synthetic polymer‐based scaffolds offer predictable and controllable degradation profiles, avoiding the batch‐to‐batch variability and undefined composition often associated with natural materials [51, 58, 59]. Through rational molecular design, synthetic polymer‐based scaffolds are evolving into versatile platforms with tunable biocompatibility and bioactivity, allowing their biological performance to be tailored for specific applications in advanced 3D cancer modeling.

Synthetic polymers thus enable controlled modulation of physicochemical properties, supporting systematic studies of mechanobiology and drug response. However, their clinical translation relies on overcoming biocompatibility challenges through advanced functionalization and hybrid designs. As engineered matrices evolve, they hold promise for creating standardized, patient‐specific platforms that bridge the gap between simplistic screens and physiological complexity.

### 3.3 Hybrid and Composite Scaffolds

Hybrid and composite scaffolds have been developed to overcome the limitations of individual material classes, such as the weak mechanical properties of natural polymers and the limited bioactivity of some synthetic systems. By combining complementary materials, these scaffolds create more physiologically relevant microenvironments for 3D tumor culture.

Composite systems that integrate poly(ethylene glycol) diacrylate (PEGDA) with natural polymers are particularly notable. PEG is widely used owing to its excellent biocompatibility, hydrophilicity, low protein adsorption, and minimal immunogenicity [60], but it lacks cell‐adhesive motifs. PEGDA provides a mechanically tunable backbone with precise control over stiffness, porosity, and crosslinking density [44]. Incorporation of bioactive natural polymers compensates for PEG's inertness. For example, GelMA introduces RGD motifs and MMP‐sensitive degradation sites, enabling cell adhesion, spreading, and protease‐driven invasion. At the same time, PEGDA reinforces the mechanical stability of the otherwise soft GelMA matrix [44, 56]. Similarly, methacrylated hyaluronic acid (HAMA) is frequently incorporated as a signaling component rather than a structural element. In PEG‐HAMA hydrogels, the synthetic PEG network maintains constant mechanical properties, whereas HAMA engages cell‐surface receptors such as CD44, thereby decoupling mechanical cues from biochemical signaling [61]. This strategy has revealed dose‐dependent roles of HA in promoting malignant phenotypes, including cell clustering and upregulation of pro‐invasive genes [57]. In PEG‐fibrinogen composite scaffolds, fibrinogen contributes native cell‐binding domains and enzymatic cleavage sites characteristic of tumor stroma, supporting diverse cancer cell morphologies [62].

PCL is another widely used structural component in composite scaffolds due to its mechanical stability and processability. However, its limited bioactivity necessitates functionalization or combination with other materials to achieve biomimetic performance [63]. For instance, PCL scaffolds infused with tyramine‐modified HA hydrogels form composite constructs in which the macroporous PCL framework provides mechanical integrity. In contrast, the HA hydrogel supplies biochemical cues through interactions with receptors such as CD44 and receptor for hyaluronan‐mediated motility (RHAMM) [59]. This dual‐porosity architecture supports cell infiltration and growth while simultaneously mimicking both the rigid tissue scaffold and the soft ECM‐rich interstitial space. Notably, PCL‐HA composite scaffolds have been shown to enrich CSC populations, offering valuable platforms for studying drug resistance and tumor recurrence.

Hybrid scaffolds exemplify the convergence of material science and tumor biology, enabling decoupled control over mechanical and biochemical cues. By combining the strengths of natural and synthetic components, these systems more faithfully replicate the dynamic and heterogeneous nature of the TME. Moving forward, rational design of composite matrices should prioritize not only mimicry but also tunability, to model stage‐specific microenvironmental evolution and therapy‐induced remodeling.

### 3.4 Decellularized Extracellular Matrix Scaffolds

Decellularized extracellular matrix (dECM) scaffolds are derived from tissues and retain multiple structural and biochemical features of the native ECM. These scaffolds are generated by removing cellular components from normal or tumor tissues using physical and chemical methods while retaining ECM architecture, composition, and bioactive factors [64, 65]. dECM can be derived from normal organs, such as the liver or lung, or patient‐derived tumor tissues [66]. The latter retains the unique molecular characteristics of that specific type of tumor ECM, including its distinctive protein composition, degree of collagen cross‐linking, and mechanical properties [67]. Cancer‐derived dECM has been shown to promote cell migration, angiogenesis, and epithelial‐mesenchymal transition (EMT), making it particularly valuable for reconstructing patient‐specific TMEs in vitro.

As natural materials, dECM scaffolds retain a broad range of tissue‐specific ECM proteins, including collagen, fibronectin, and laminin, as well as glycosaminoglycans and growth factors, while exhibiting low immunogenicity [43, 68, 69]. These complex biochemical cues regulate cell proliferation, migration, differentiation, and survival more effectively than simplified hydrogels composed of one or two components. Moreover, preserved tissue architecture provides structural and mechanical guidance that promotes physiologically relevant 3D cell morphologies and dynamic cell–ECM interactions.

For in vitro tumor modeling, dECM can be processed into recellularized scaffolds, injectable hydrogels, or bioinks for 3D bioprinting [70]. However, dECM‐based materials often suffer from limited mechanical tunability, component loss during decellularization, and poor structural stability [71]. To address these challenges, strategies such as embedding dECM within synthetic or natural polymer matrices [72, 73], chemical crosslinking of ECM components [74], and introduction of modifiable functional groups have been employed [73, 74].

dECM scaffolds provide a biologically relevant approach for preserving multiple tissue‐specific ECM complexity and constructing patient‐derived tumor models. However, their translational utility is constrained by batch variability, limited mechanical tunability, and scalability challenges. Innovations in processing, stabilization, and integration with tunable hydrogels will be essential to harness dECM's full potential for personalized oncology and high‐throughput drug testing.

### 3.5 Effects of Biomaterials on Cancer Cells

Biomaterials used in 3D culture systems have a profound impact on the behavior of cancer cells. Careful selection of materials that reflect the pathophysiological characteristics and ECM composition of specific tumors is therefore essential for constructing predictive and biomimetic models. HA is a nonsulfated, anionic glycosaminoglycan and a major component of the ECM in normal brain tissue [75, 76]. In glioblastoma (GBM), alterations in local HA abundance are closely associated with tumor progression [61, 77]. HA activates signaling through CD44 and RHAMM on GBM cells, thereby promoting migration and invasion [59, 76, 77]. These disease‐relevant interactions make HA‐based hydrogels particularly suitable for in vitro GBM modeling. However, the biological effects of HA depend strongly on its concentration and molecular weight. Although increased HA accumulation is generally associated with tumor progression, excessively high concentrations may produce opposing effects [78]. Moreover, HA molecular weight also influences GBM behavior. High‐molecular‐weight HA resembles the predominant form in normal brain tissue and can promote CD44 clustering, whereas low‐molecular‐weight HA has been associated with enhanced stemness and therapeutic resistance in glioma stem cells (GSCs) [79, 80]. Therefore, the concentration, molecular weight, integrin‐binding peptides, and mechanical properties of HA should be carefully considered when designing biomimetic 3D GBM models. For example, in collagen I/III–HA composite hydrogels, increasing the HA content raised the elastic modulus from approximately 300 to 2065 Pa, enabling the matrices to span a mechanically relevant range for brain tissue. At low HA concentrations (≤0.5 wt%), patient‐derived OSU‐2 GBM cells exhibited spread, spindle‐shaped morphologies and mesenchymal‐like migration, similar to those observed in collagen‐only matrices. By contrast, increasing HA concentrations induced a transition toward a rounded morphology, progressively reduced migration speed at concentrations of ≥0.2 wt%, and ultimately abolished detectable migration at higher HA levels. These findings demonstrate that HA content can markedly regulate GBM morphology and migration in 3D matrices [81].

Increasingly, studies have systematically compared the effects of different biomaterials on tumor cells to guide rational scaffold design. For example, comparative analyses of collagen, Matrigel, alginate, and agarose hydrogels demonstrated that materials with higher stiffness or elasticity, such as high‐concentration collagen and agarose, more effectively maintain malignant phenotypes of osteosarcoma (OS) cells in 3D culture [82]. This effect is attributed to activation of integrin‐focal adhesion signaling pathways by elastic matrices that mimic pro‐tumorigenic mechanical cues in vivo. At the same time, reduced adhesive constraints may facilitate cell migration and metastasis.

These findings underscore that biomaterial selection is not merely a structural consideration but a decisive factor in modeling tumor phenotypes and therapeutic responses. As the field progresses, systematic comparisons across material classes should be integrated with omics and functional readouts to establish predictive design principles. Ultimately, biomaterial‐driven models must evolve from passive scaffolds to active instructors of tumor behavior, mirroring the dynamic reciprocity between cancer cells and their ECM in vivo.

## 4 Recapitulating the Tumor Microenvironment Through Scaffold Design

### 4.1 Mimicking the Physical TME

The ECM is a central component of the TME, exerting profound control over cellular behavior. In addition to biochemical alterations, the biophysical properties of the ECM are equally critical determinants of tumor initiation, progression, and metastasis. The physical properties of the ECM include stiffness, solid stress, fluid pressure, and tissue microstructure. Recapitulating these physical properties in vitro is therefore essential for investigating tumor behaviors in 3D environments and for predicting cellular responses to mechanical cues and pharmacological interventions encountered during cancer progression (Figure 2).

**FIGURE 2:**
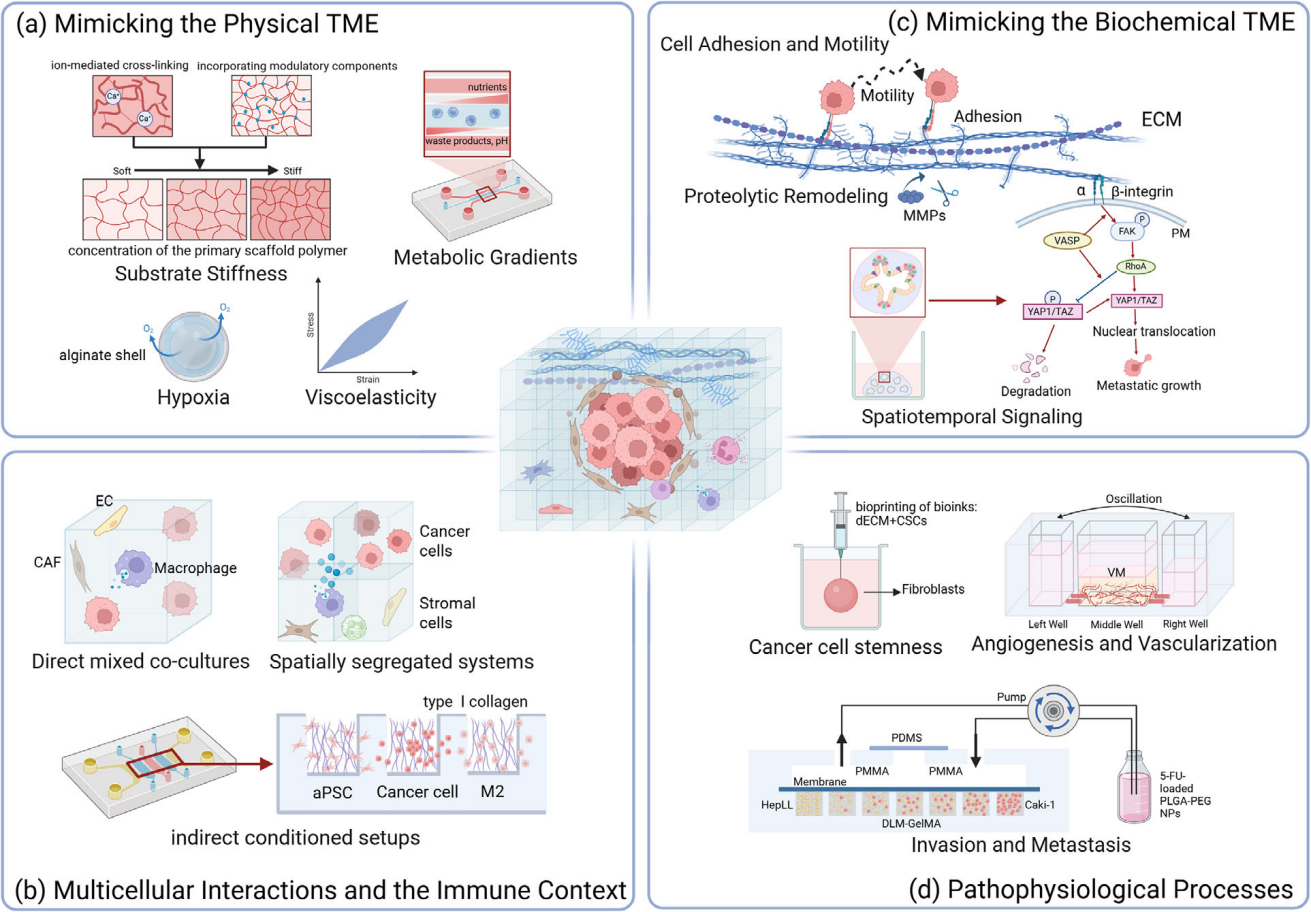
This figure summarizes strategies for engineering advanced 3D biomimetic scaffolds that recapitulate key features of the TME. (a) Physical TME mimicry: Matrix stiffness is precisely tuned from soft to rigid. Critical mechanical and microenvironmental cues, including hypoxia, metabolic gradients, and viscoelastic properties, are engineered; (b) Reconstruction of multicellular interactions and the immune microenvironment: Direct mixed co‐culture, spatially organized co‐culture, and indirect conditioned setups are used to integrate tumor cells with stromal, immune, and ECs; (c) Biochemical TME mimicry: Scaffold design focuses on reproducing cell–ECM adhesion and migration, MMP‐mediated proteolytic remodeling, and mechanotransduction pathways. Key spatiotemporal signaling networks, such as the integrin–FAK–RhoA–YAP/TAZ axis, are incorporated; (d) Modeling key tumor pathological processes: The resulting platforms enable the study of dynamic processes, including maintenance of cancer stemness, angiogenesis and vascularization, as well as tumor invasion and metastasis, thereby providing physiologically relevant in vitro models for mechanistic investigation and therapeutic evaluation. EC, endothelial cell; CAF, cancer‐associated fibroblast; PSC, pancreatic stellate cells; MMP, matrix metalloproteinases; M2, M2 macrophages; CSC, cancer stem cell; VM, vasculogenic mimicry.

#### 4.1.1 Substrate Stiffness

ECM stiffness is a critical biomechanical property that refers to the resistance of the ECM to deformation under mechanical stress. The stromal stiffness of solid tumors within the body often differs from that of normal tissues [24]. Dysregulation in the synthesis, deposition [98], remodeling [99], cross‐linking [98], and enzymatic modification of the ECM is a key driver of fibrosis and consequent matrix stiffening. Accumulating evidence indicates that matrix stiffness can regulate cancer cell proliferation [100, 101], migration [102], chemotherapeutic response [103], as well as stem cell‐like properties [104, 105]. Consequently, substrate stiffness represents an important design parameter in 3D tumor models. Several established strategies are available to fabricate and modulate matrix stiffness in vitro: (i) varying the concentration of the primary scaffold polymer, (ii) incorporating modulatory components without altering the base matrix composition, and (iii) employing ion‐mediated cross‐linking.

A commonly used approach involves adjusting the concentration of a single polymer. Increasing polymer concentration enhances network density and cross‐linking, reduces pore size, and improves structural integrity, resulting in concentration‐dependent stiffening. In hydrogel systems, for example, increasing GelMA concentration from 5% to 15% elevates the storage modulus from approximately 1 kPa to over 130 kPa, spanning mechanical environments from soft tissues to rigid OS niches [106]. Similarly, increasing collagen concentration from 3 to 6 mg/mL raises the Young's modulus of collagen hydrogels from roughly 1 to 14 kPa [107]. Despite being simple to implement, this approach offers limited control over matrix mechanics, considering the changes in polymer concentration simultaneously influencing pore structure, ligand availability, and mass transport.

Matrix stiffness can also be finely tuned by introducing secondary components while maintaining the primary matrix composition. A common strategy involves blending a natural biomaterial with a synthetic polymer that provides additional cross‐linking sites or forms an interpenetrating polymer network. For instance, GelMA alone exhibits limited mechanical stability during long‐term culture. Incorporation of PEGDA, a non‐adhesive synthetic polymer, acts as a multifunctional cross‐linker that reinforces the hydrogel network. By modulating the GelMA‐to‐PEGDA ratio, stiffness can be precisely adjusted within a range of 7–52 kPa [108]. This design therefore provided a broader stiffness range while preserving comparable adhesive ligand availability.

Ion‐mediated cross‐linking offers another powerful means of stiffness modulation, particularly for anionic polymers such as alginate and collagen. By holding the concentration of the primary matrix constant and varying only the concentration of cross‐linking ions such as Ca^2+^, hydrogels with a broad stiffness range can be generated while preserving identical biochemical cues and microarchitecture. This approach is especially valuable for modeling intratumoral mechanical heterogeneity observed in cancers such as breast cancer (BC) and hepatocellular carcinoma (HCC) [105, 109], where stiffness often increases from the tumor core toward the invasive front. For example, in a gelatin/alginate hydrogel system designed to mimic the breast TME, stiffness was tuned from approximately 2 kPa (core‐like) to 17 kPa (periphery‐like) by increasing calcium chloride concentration from 3.75 to 30 mM [109].

Beyond these conventional approaches, alternative strategies have been developed to modulate matrix stiffness. Self‐assembling peptide nanofibrous matrices, such as RAD16‐I, allow stiffness control through peptide concentration while simultaneously adsorbing serum proteins to support cell adhesion. Their low biodegradability maintains relatively stable mechanical conditions during culture, although the absence of intrinsic receptor‐binding motifs limits the recapitulation of specific cell–matrix interactions [27]. Composite hydrogels combining adipose‐derived decellularized matrix with silk fibroin exhibit time‐dependent stiffening, increasing from ∼10–11 to ∼25 kPa over 21 days due to progressive β‐sheet formation within silk fibroin chains [110]. Electrospinning‐based approaches enable stiffness regulation through fiber composition; for example, coaxial electrospinning of PCL cores and gelatin shells allows modulation of the macroscopic Young's modulus by adjusting the polymer flow rate ratios [111]. In a bioinspired strategy, genetic modulation of lysyl oxidase expression in cancer cells has been used to generate tumors with distinct collagen cross‐linking levels in vivo. Decellularization of these tissues yields ECM scaffolds that preserve native biochemical composition and architecture while exhibiting intrinsically programmed mechanical properties [112]. The use of xenograft‐derived matrices strengthens the level of biological validation relative to purely in vitro stiffness‐tuning systems, although the multistep workflow is costly and limits throughput.

Tumor stiffness is closely linked to the composition and organization of the ECM. Accordingly, in vitro models should reproduce the characteristic stiffness ranges associated with specific tumor types and stages of tumor progression. In glioma, tissue stiffness generally increases with histological grade, with normal brain tissue being the softest (Young's modulus, E; 0.01–0.18 kPa), while low‐grade gliomas (E; 0.05–1.4 kPa) and GBMs (E; 0.07–13.5 kPa) exhibit generally higher upper limits and broader stiffness ranges [113]. These grade‐dependent mechanical differences highlight the importance of recapitulating cell‐ECM interactions in vitro GBM models. BC exhibits a distinct, stroma‐dominated pattern of ECM stiffening. The BC tissue exhibits higher stiffness (elastic modulus of 1–4 kPa) than the normal mammary gland (elastic modulus of 0.15–0.20 kPa). Tumor‐associated stroma (E: 0.4–1 kPa) is also stiffer than normal stroma (E: 0.2 kPa) [114]. The stiffening of the tumor‐associated ECM is primarily attributed to increased collagen deposition, lysyl oxidase‐ and transglutaminase‐mediated collagen crosslinking, and non‐enzymatic glycation [115]. These compartment‐specific mechanical differences indicate the importance of recapitulating both whole‐tumor stiffness and the distinct mechanical properties of normal and tumor‐associated stroma when developing in vitro BC models. Compared with GBM and BC, which originate from relatively compliant brain and mammary tissues, respectively, OS arises within a substantially stiffer bone‐associated microenvironment. In specific hydrogel‐based models, matrices with a stiffness of approximately 55 kPa have been shown to promote OS cell growth, spheroid formation, and migration [116]. These findings highlight the importance of tailoring matrix stiffness to both the mechanical properties of the tissue of origin and the pathological characteristics of the TME.

Intratumoral matrix stiffness is highly heterogeneous [117]. Tissues from BC and HCC are typically stiffer than their normal counterparts and display a gradient of increasing stiffness from the tumor core to the invasive margin [105, 109]. To recapitulate this mechanical landscape, gelatin/alginate hydrogels with calcium‐dependent cross‐linking have been engineered to represent discrete stiffness zones corresponding to core, intermediate, and peripheral tumor regions [109]. Despite these advances, many existing 3D culture models fail to reproduce the absolute stiffness values or spatial heterogeneity present in native tumors. There is therefore a pressing need for in vitro platforms that can precisely mimic tissue‐specific and spatiotemporally resolved stiffness landscapes across different cancer types.

These approaches highlight the central role of matrix stiffness in directing cancer cell fate and therapeutic response. However, most current models still struggle to replicate the spatial and temporal heterogeneity of stiffness observed in vivo. Future platforms must integrate dynamic stiffness modulation and patient‐specific mechanical profiling to better evaluate how localized matrix remodeling influences progression and treatment resistance.

#### 4.1.2 Hypoxia and Metabolic Gradients

Restricted vascularization and high metabolic demand in solid tumors give rise to hypoxia [118], nutrient depletion, waste accumulation [119], and pronounced pH gradients [120, 121]. As a result, tumor cells located near blood vessels receive sufficient oxygen and nutrients to proliferate, forming a viable peripheral region, whereas cells in poorly perfused central regions undergo nutrient starvation and death, leading to the formation of a necrotic core [118, 122]. Accurately modeling hypoxic conditions in vitro is therefore essential for studying tumor progression, metabolic adaptation, and therapy resistance.

Hypoxia is a ubiquitous hallmark of solid tumors [122], yet oxygen levels in conventional 3D cultures are often poorly controlled and spatially heterogeneous due to diffusion limitations and cell‐dependent oxygen consumption [118]. Emerging 3D culture technologies enable more precise reconstruction of hypoxic TMEs. One strategy involves physically restricting oxygen diffusion by engineering core–shell architectures. For example, alginate‐based systems consisting of a cellular core surrounded by an additional alginate shell create an effective diffusion barrier, establishing a stable hypoxic niche (∼1.3% O_2_) within the core under normoxic culture conditions, as confirmed by elevated hypoxia‐inducible factor 1 alpha (HIF‐1α) expression [89].

A more broadly applicable approach exploits the intrinsic relationship between construct size and oxygen diffusion. Hydrogel microspheres with defined diameters can be fabricated to generate predictable hypoxic cores. Electrostatic encapsulation produces alginate beads with narrow size distributions, and beads with radii exceeding ∼500 µm spontaneously develop hypoxic centers whose dimensions scale with bead size. This size‐dependent model closely mimics physiological oxygen gradients in avascular tumor regions [123]. The core–shell design enables fine control over oxygen transport by varying shell thickness and matrix density, thereby supporting stable yet tunable hypoxic conditions. By comparison, size‐dependent diffusion models generate physiological oxygen gradients through simple adjustment of construct dimensions. Their compatibility with technologies such as 3D bioprinting further offers advantages in throughput and reproducibility [124]. Together, these strategies provide robust and reproducible platforms for investigating hypoxia‐driven signaling, metabolic reprogramming, and therapeutic responses under physiologically relevant low‐oxygen conditions.

Beyond oxygen gradients, the TME exhibits substantial metabolic heterogeneity, characterized by spatial variations in nutrient availability, waste accumulation, and pH. Recapitulating these gradients is critical for understanding cancer cell plasticity and drug resistance. Advanced 3D culture platforms, particularly microfluidic systems, have emerged as powerful tools for this purpose through two complementary strategies. The first strategy relies on the formation of endogenous gradients driven by cellular metabolism and diffusion constraints. In the colon cancer microfluidic model, a central chamber containing tumor cell‐laden collagen hydrogels is flanked by perfused channels that mimic vasculature. Carefully designed chamber dimensions and diffusion ports enable slow, uniform nutrient penetration, while high cellular metabolic activity generates gradients of nutrients, lactate, and pH. Cells near the perfusion channel remain well nourished, whereas distal cells experience nutrient deprivation and acidosis [11]. This endogenously generated microenvironment more faithfully reproduces the pathological niche of solid tumors. Nonetheless, nutrient depletion, metabolic waste accumulation, and hypoxia often arise concurrently, making it difficult to isolate the contribution of each individual factor. The second strategy involves the controlled imposition of exogenous chemical gradients. By contrast, exogenous approaches actively impose defined chemical gradients, offering greater programmability and experimental control. In open, polydimethylsiloxane (PDMS)‐free microfluidic systems, tumor cells encapsulated in hybrid hydrogels directly interface with longitudinal fluidic channels. By introducing chemokines at one end and applying aspiration at the other, stable and tunable concentration gradients can be established across millimeter‐scale hydrogels through the combination of laminar flow and diffusion. Despite these advances, accurately capturing the full spatial metabolic and phenotypic heterogeneity of tumors in vitro remains a significant challenge [125].

Accurately modeling hypoxia and metabolic gradients is therefore essential for dissecting therapy resistance mechanisms rooted in metabolic adaptation. While current systems successfully generate stable oxygen and nutrient gradients, they often lack the dynamic coupling between vascular perfusion, cellular consumption, and matrix remodeling seen in vivo. Next‐generation models should aim to integrate real‐time metabolic imaging with spatially resolved omics to decode how gradient‐driven heterogeneity shapes tumor evolution.

#### 4.1.3 Viscoelasticity

Tumor viscoelasticity refers to a composite mechanical property exhibited by tumor tissue and its ECM, encompassing both elasticity, the ability to recover from deformation, and viscosity, the ability to resist deformation and dissipate energy. Stress relaxation is an important parameter that describes this behavior [126]. Despite its physiological relevance, the role of viscoelasticity in regulating tumor development and therapeutic response within 3D microenvironments remains poorly understood.

To address this gap, dynamically cross‐linked hydrogel systems have been developed to independently tune viscoelastic properties. For example, PEG‐based hydrogels formed via dynamic hydrazone cross‐linking enable precise control of stress relaxation behavior. Incorporation of a constant concentration of type I collagen creates an interpenetrating network that preserves consistent biochemical adhesion cues. By adjusting the molar ratio of alkyl‐hydrazone to benzyl‐hydrazone bonds, stress relaxation can be systematically regulated while maintaining comparable stiffness, thereby mimicking the viscoelastic properties of brain tissue. This platform has enabled detailed investigation of viscoelastic effects on 3D GBM cell behavior [127].

Nevertheless, systematic studies focusing on viscoelasticity as a key mechanical regulator in 3D tumor models remain limited. Future work should expand material platforms by integrating diverse dynamic chemistries and biomimetic designs to elucidate tumor‐specific responses to viscoelastic microenvironments. Such efforts will be essential for developing more physiologically relevant in vitro models for drug screening and optimizing therapeutic strategies.

### 4.2 Mimicking the Biochemical TME

The ECM is primarily composed of core matrisome proteins, including diverse collagens, glycoproteins, and proteoglycans, as well as matrisome‐associated molecules. Collagen, the most abundant fibrous ECM protein, along with elastin, provides structural integrity, whereas glycoproteins and proteoglycans mainly regulate cell adhesion, signaling, and growth factor presentation [24]. The organization and biochemical composition of the ECM critically modulate cancer cell genotypes and phenotypes and, consequently, multiple hallmarks of cancer. These effects can be systematically dissected using 3D culture models. Accordingly, functionalized hydrogels incorporating defined bioactive motifs have been developed to interrogate how individual ECM components regulate specific cancer cell behaviors and biological processes.

#### 4.2.1 Cell Adhesion and Motility

In vivo, cancer cell attachment to the ECM is mediated predominantly by adhesion receptors, particularly integrins, which link extracellular ligands to the cytoskeleton and intracellular signaling networks. Remodeling of these adhesions, including loss or reprogramming of adhesion molecules, accompanies EMT and licenses tumor cell migration and invasion [23]. In collagen/HA composite hydrogels, GBM cell migration decreases with increasing HA content, indicating a concentration‐dependent suppression of motility by HA [81]. In contrast, in a 3D ovarian cancer model, type I collagen enhances cell motility by upregulating MMPs and α5β1 integrin, highlighting pro‐invasive crosstalk between fibrillar collagen and integrin signaling [128].

Ligand density is a critical design parameter and must be optimized within a narrow window. In galactosylated sodium alginate scaffolds, pro‐adhesive effects diminish above 2% (w/v), consistent with excessive ligand presentation and/or network densification that restricts cell spreading [129]. Beyond structural cues, ECM‐bound signaling molecules, such as epidermal growth factor (EGF), further regulate migratory capacity through receptor tyrosine kinase pathways [35, 55]. To ensure reproducibility and precise control of biochemical signals, synthetic matrices are frequently functionalized with defined peptide ligands. For example, a recombinant spider‐silk scaffold engineered with the fibronectin‐derived RGD motif self‐assembles under physiological conditions, enhances integrin‐mediated adhesion, promotes homogeneous cell distribution, and upregulates adhesion‐associated genes, including integrin β1 and focal adhesion kinase (FAK) [130].

Synthetic and natural ECM analogues enable controlled presentation of selected adhesive ligands, but they rarely reproduce the coordinated biochemical and mechanical cues that govern tumor cell motility in native tissues. Tissue‐derived dECM partly overcomes this limitation by retaining organ‐specific biochemical and structural cues that regulate tumor cell adhesion and motility [131]. Considering that dECM hydrogel processing can compromise mechanical integrity, it is often combined with tunable polymers to support cell adhesion, migration, and invasion [132].

These findings underscore that cell–ECM adhesion is not merely a structural feature but a dynamic signaling node that can be engineered to probe invasion mechanisms. However, adhesion ligand presentation requires careful optimization to avoid paradoxical effects on motility. Future designs should incorporate spatially patterned adhesive cues to better mimic the heterotypic adhesion landscapes found in invasive fronts.

#### 4.2.2 Proteolytic Remodeling

Proteolytic degradation, post‐translational chemical modifications, and force‐mediated physical reorganization drive ECM remodeling. In vivo, tumor cells actively recruit and activate stromal populations that deposit and remodel ECM within the TME [24, 128, 133]. In vitro, these processes are modeled by incorporating natural ECM fractions into 3D scaffolds or by using compositionally defined hydrogels to isolate the effects of specific matrix components on cancer cell behavior [57, 128, 134].

Fibrillar collagen I is a dominant feature of desmoplastic stroma and is frequently associated with enhanced tumor cell survival and metastatic dissemination across multiple cancer types. When ovarian cancer cells are embedded within collagen I matrices, motility and invasion are promoted through upregulation of MMPs and α5β1 integrin [128]. In 3D systems, ECM‐associated enzymes such as MMPs, disintegrins and metalloproteinases (ADAMs), and cathepsin activity serve as a functional indicator of ongoing matrix degradation and turnover. Whereas interstitial collagen in healthy tissues is essentially isotropic, many tumors exhibit densely bundled and anisotropically aligned collagen fibers that provide preferential migration tracks. Microfluidic platforms exploit this feature to control collagen fiber alignment; compared with isotropic or agarose‐composite matrices, aligned collagen bundles enhance cancer cell outgrowth and accelerate directed migration [83, 133]. Proteolytic remodeling also evolves during tumor progression and metastasis. Decellularized BC ECM derived from low‐ versus high‐metastatic lesions identifies collagen IV as a key determinant of matrix stiffening and increased cell migration, underscoring the role of specific basement membrane components in regulating TME mechanics and invasiveness [135].

Proteolytic remodeling thus serves as both a biomarker and a functional driver of tumor progression. While current models capture protease activity and matrix degradation, they often overlook the reciprocal feedback between ECM cleavage, fragment generation, and pro‐tumor signaling. Incorporating degradable motifs with reporting capabilities will help visualize and quantify spatiotemporal remodeling dynamics in vitro.

#### 4.2.3 Spatiotemporal Signaling

Alterations in ECM composition frequently increase the local availability of matrix‐bound growth factors and cytokines, thereby driving tumor behavior toward progression. These biochemical signals are transmitted through cell‐surface receptors, including integrins, collagen‐binding discoidin domain receptors, laminin‐binding dystroglycans, and HA receptors such as CD44, which collectively coordinate outside‐in signaling [22, 99]. Matrigel‐based 3D spheroid models have been widely used to capture ECM‐induced β1‐integrin‐FAK‐YAP/TAZ signaling. In gastrointestinal cancer cells, the actin regulator vasodilator‐stimulated phosphoprotein (VASP) is required for activation of this pathway. VASP knockdown suppresses FAK phosphorylation, reduces YAP1/TAZ protein levels, and restricts 3D spheroid growth. Mechanistically, VASP promotes ECM‐dependent β1‐integrin activation and facilitates YAP1/TAZ dephosphorylation downstream of RhoA, thereby enhancing YAP1/TAZ protein stability. Consistently, VASP depletion in vivo reduces liver metastasis, supporting the relevance of ECM‐dependent β1‐integrin‐FAK‐YAP/TAZ signaling to metastatic potential [136].

Defined ECM ligands can be incorporated into 3D matrices to program receptor usage and signaling dynamics. Photopolymerizable hydrogels presenting collagen‐mimetic GFOGER and fibronectin/vitronectin‐mimetic RGDS motifs promote β1‐integrin‐dependent BC spheroid outgrowth. GFOGER‐rich matrices exhibit density‐dependent proliferation and transcriptional enrichment of cell–matrix signaling and motility pathways [22]. In triple‐negative breast cancer (TNBC), COL8A1, a subunit of collagen VIII, is highly expressed and hypoxia‐inducible. Exogenous COL8A1 supplementation in 3D spheroids activates FAK/Src signaling and accelerates growth, whereas pharmacological inhibition of FAK with defactinib suppresses spheroid expansion without overt cytotoxicity [137].

Beyond native ECM components and peptide ligands, emerging protein‐tunable scaffolds enable causal interrogation of individual matrix proteins. For example, hyaluronan‐based hydrogels assembled using SpyTag/SpyCatcher chemistry can be modularly decorated with specific signaling proteins at defined densities. Matrix‐tethered EpCAM alone is sufficient to disrupt non‐malignant mammary epithelial spheres in 3D, illustrating how a single ECM‐anchored cue can reprogram epithelial organization [138]. Compared with the immobilization of full‐length proteins, incorporating short ECM‐mimetic ligands into 3D matrices is better suited to quantitative control over ligand identity, density, and combinatorial presentation. Alternatively, short peptide motifs cannot preserve the full conformational complexity of native ECM proteins. Beyond mediating cell adhesion, the ECM also serves as a reservoir for growth factors and cytokines, whose sequestration and release are regulated by matrix remodeling and interstitial flow. Accordingly, scaffolds that preserve or deliberately engineer this reservoir are essential for physiological in vitro modeling. A patient‐derived dECM hydrogel, termed CologEM, was developed as a biomimetic scaffold for colorectal cancer modeling with preserved collagen architecture and endogenous biochemical components. This architecture provides a broad spectrum of ECM‐associated bioactive molecules, including interleukins, chemokines, growth factor‐associated proteins, and ECM‐remodeling regulators. Functionally, HCT‐116 and HCT‐15 colorectal cancer cells cultured within CologEM exhibited enhanced EMT‐associated gene expression and resistance to 5‐fluorouracil treatment. Collectively, this ECM‐derived hydrogel demonstrates how engineered dECM scaffolds can recapitulate tumor–matrix interactions in advanced 3D culture models. Nonetheless, translating such systems into clinically predictive platforms remains challenging, as donor‐dependent ECM heterogeneity and processing‐related batch effects may compromise reproducibility [139].

Controlled presentation of ECM‐bound signals could facilitate analysis of pathway activation in physiologically relevant contexts. Yet, the dynamic reciprocity among matrix remodeling, growth factor release, and cellular feedback remains challenging to recapitulate. Platforms that integrate real‐time signaling biosensors with tunable ligand presentation will be essential to decode the temporal evolution of ECM‐driven oncogenic signaling.

### 4.3 Modeling Multicellular Interactions and the Immune Context

The TME is continuously shaped by tumor cells and diverse non‐malignant populations, including immune cells, cancer‐associated fibroblasts (CAFs), MSCs, cancer‐associated adipocytes (CAAs), endothelial cells (ECs), pericytes, and organ‐specific tissue‐resident cells. These host cells are either recruited from adjacent tissues or reprogrammed within the tumor by tumor‐derived factors. Conversely, non‐neoplastic cells communicate with tumor and microenvironmental components through cytokines, soluble mediators, and direct cell–cell contacts, thereby promoting tumor initiation, invasion, metastasis, and recurrence [1, 140]. The interaction between non‐neoplastic cells and tumor cells is widely researched in vitro. Basically, three canonical formats are used to coculture them: (i) spatially segregated systems that position tumor and non‐neoplastic cells in distinct layers, channels, or scaffold compartments to recreate tissue organization and gradients; (ii) direct mixed co‐cultures that embed both cell types within the same matrix and medium to capture juxtacrine and short‐range paracrine signaling; and (iii) indirect conditioned setups that expose tumor cells to non‐neoplastic cell secretomes, perfusates, or decellularized matrices to isolate soluble and matrix‐encoded effects.

Spatially segregated systems enable paracrine interactions between cell types while maintaining distinct localization for facile visualization [141, 142, 143]. Qiao et al. developed a 3D co‐culture platform to examine interactions between human umbilical vein endothelial cells (HUVECs) and mouse breast CSCs. In this bilayer system, CSCs were cultured in alginate‐based hydrogel microcapsules in the upper layer, while stromal cells were encapsulated in alginate‐chitosan hydrogels in the lower layer. Co‐culture with HUVECs induced marked upregulation of CD44 and MMP9 in tumor spheroids, indicating enhanced stemness. This platform provides a simple architecture with clearly defined cellular compartments but overlooks the contributions of perfusion, the endothelial barrier, and direct cell–cell contact in vivo. Spatial separation is particularly powerful for modeling early, noninvasive tumor‐immune crosstalk, when lesions remain epithelial and immune cells are recruited from the vasculature [142]. In a related microfluidic design, a bilayer GelMA construct confined BC cells and monocytes within an inner gel, while HUVECs formed an outer endothelial sleeve. Activated T cells delivered through perfused channels were required to extravasate across this engineered endothelium, closely mimicking immune access to nascent tumors. Under these conditions, spheroid tumors recruited more T cells than dispersed cancer cells, and monocytes further amplified T‐cell infiltration by increasing secretion of CCL4, CCL5, CCL11, and CXCL8 [144]. The sophisticated bilayer GelMA microfluidic platform recapitulates sequential T‐cell adhesion, transendothelial extravasation, and tumor infiltration under perfusion. Despite this, its technically demanding fabrication and operation may limit reproducibility and broader adoption. Using a similar gel‐in‐gel configuration, Buonvino et al. investigated how human fibroblasts modulate the migration of MDA‐MB‐231 BC cells [145].

Direct mixed co‐culture systems embed tumor and non‐neoplastic cells within the same 3D matrix, allowing continuous bidirectional crosstalk and detailed analysis of dynamic cellular behaviors [146, 147, 148, 149, 150, 151, 152]. In one example, PT45 pancreatic cancer cells were co‐cultured with either normal fibroblasts or CAFs on biodegradable gelatin microcarriers in a spinner bioreactor. Exposure to tumor cells activated fibroblasts toward a CAF‐like phenotype, characterized by upregulation of α‐smooth muscle actin (α‐SMA), PDGFRβ, and a broad desmoplastic ECM gene program. Fibroblasts deposited a collagen‐rich matrix that replaced the original scaffold and promoted invasive tumor patterning. Notably, this dual remodeling was absent in tumor monocultures, which failed to generate organized ECM or expand over time, underscoring the requirement for tumor‐stroma co‐residence to recapitulate pancreatic ductal adenocarcinoma (PDAC)‐like matrix accumulation. Wu et al. further constructed 3D full‐thickness models of vulvar squamous cell carcinoma by seeding tumor cells (A431 or HTB117) atop dermal matrices populated with defined fibroblast subtypes—papillary fibroblasts (PFs), reticular fibroblasts (RFs), or patient‐derived CAFs. Replacement of PFs with RFs or CAFs increased tumor expression of E‐cadherin, vimentin, and K19, consistent with enhanced proliferation and stemness. Strikingly, RFs were reprogrammed by tumor cells, beginning to express CAF markers such as α‐SMA and COL11A1, revealing an in‐model RFs‐to‐CAF transition not observed with papillary fibroblasts [146]. By recapitulating sequential T‐cell adhesion, transendothelial extravasation, and tumor infiltration under perfusion, this sophisticated bilayer GelMA microfluidic platform offers enhanced physiological and clinical relevance. Its technically demanding fabrication and operation may nevertheless limit reproducibility and broader adoption.

Innate immune cells and tumor cells also engage in highly plastic, bidirectional interactions that can either restrain or promote malignancy. Within the TME, cytokines, growth factors, and damage‐associated molecular patterns (DAMPs) released by stressed or necrotic tumor cells can polarize macrophages, monocytes, and other myeloid cells toward tumor‐supportive states. Conversely, pattern‐recognition receptor and Toll‐like receptor sensing of DAMPs can enhance phagocytosis and stimulate adaptive immune responses. Cytotoxic innate immune mechanisms can directly eliminate tumor cells via perforin/granzyme release or death‐receptor signaling, processes that are finely tuned by the balance of activating and inhibitory ligands on cancer cells [153]. Direct mixed co‐cultures are therefore particularly informative for capturing the full spectrum of contact‐dependent and paracrine interactions governing tumor‐innate immune crosstalk in vitro [154, 155, 156, 157].

In indirect conditioned setups, tumor cells are not co‐localized with stromal cells. Instead, they are exposed to stromal‐derived cues, such as conditioned media, stromal ECM, or cytokine‐containing perfusates. This approach enables the evaluation of soluble and matrix‐encoded signals without confounding direct cell–cell contact [158, 159]. For example, an openable, multilayer PDAC on‐a‐chip platform was engineered in which pancreatic stellate cells (PSCs) embedded in type I collagen generated a desmoplastic stromal niche, while PANC‐1 tumor cells were seeded in an adjacent, diffusively connected channel defined by the TANDEM microfluidic architecture. The two cell types are not intermingled but communicate through the controlled diffusion of soluble factors and matrix‐remodeling products, allowing the tumor cells to be exposed to PSC‐conditioned stroma without the confounding effect of direct contact. PSCs upregulated activation markers and produced locally aligned, densified collagen in response to tumor‐derived signals. Analysis of conditioned media by mass spectrometry revealed stromal metabolic signatures consistent with in vivo disease [159]. However, because stromal‐tumor communication in vivo is inherently bidirectional, one‐way conditioned media capture only part of this feedback loop [157, 160, 161]. Addressing this limitation, Jang et al. established diffusively connected microfluidic channels to co‐culture pancreatic tumor spheroids (PANC‐1), naïve PSCs, and THP‐1‐derived M2 macrophages. Within 5 days, tumor‐derived cues were sufficient to activate PSCs and to polarize THP‐1/M0 cells toward CD68+/CD206+ TAM‐like states. In turn, activated PSCs and M2 macrophages induced tumor spheroids to upregulate vimentin, TGF‐β1, and CTGF, and to adopt protrusive and invasive morphologies. This bidirectional circuit also enhanced stromal cell migration toward the tumor compartment, with activated PSCs exerting the strongest promigratory effect [160].

Despite these advances, most 3D tumor‐stroma co‐culture models capture only a single snapshot of crosstalk, implicitly assuming that interactions remain constant throughout progression from in situ disease to invasive carcinoma. This assumption poorly reflects biological reality: stromal signaling is stage‐dependent, fibroblasts can switch between trophic and suppressive roles, and endocrine sensitivity or drug responsiveness may drift as paracrine factors accumulate [140]. To address temporal dynamics, a paper‐based BC model was developed to mimic discrete stages of disease progression. Estrogen receptor α (ERα)‐positive T47D‐KBluc cells were embedded in collagen I and incorporated into wax‐patterned paper scaffolds, which were then placed into standard 96‐well plates. Normal mammary fibroblasts were omitted, positioned only at the bottom of the well, or co‐embedded with tumor cells to sequentially model a normal duct with stromal separation, ductal carcinoma in situ, and invasive ductal carcinoma. This system revealed a progressive attenuation of ERα transactivation as fibroblasts were added, brought into closer proximity, or increased in number, an effect primarily mediated by fibroblast‐derived IL‐6 [162]. Importantly, the integration of wax‐patterned paper scaffolds with standard 96‐well plates indicates strong potential for scalable, high‐throughput applications.

Previous studies have shown that both the type of cancer cells used and the co‐culture configuration adopted in 3D scaffold systems can substantially affect experimental quality and applicability. The composition and relative abundance of epithelial, endothelial, immune, and stromal cells vary considerably across the TME. Therefore, a uniform cellular configuration is unlikely to be appropriate for all co‐culture systems. Table 3 summarizes the nonmalignant, nonimmune microenvironmental cell types represented in different cancers, with emphasis on their biological characteristics, functional roles within the TME, and representative 3D scaffold‐based co‐culture platforms. Accordingly, cell types should be selected according to the specific hypothesis and research question, with careful consideration of their proportions, spatial organization, and mode of co‐culture.

**TABLE 3:** The nonmalignant, nonimmune microenvironmental cell types represented in different cancers, with emphasis on their biological characteristics, functional roles within the TME, and representative 3D scaffold‐based co‐culture platforms.

Tumor type	Stromal cell	Description	Co‐culture systems	References
GBM	Astrocytes	Astrocytes are the endogenous native cells most phenotypically like the majority of GBM cells. They have been linked to promoting the invasion of CD133+ GSCs by creating a microenvironment rich in cytokines and chemokines. The interaction between astrocytes and microglial cells creates an immunosuppressive environment.	A GelMA‐GMHA bioink: patient‐derived GSCs, astrocytes, and neural stem cells, with or without macrophages.	[163, 164, 165, 166, 167]
	Pericytes	Pericytes are perivascular cells located abluminal to the ECs that support vascular structure and function, maintain the blood‐brain barrier, and contribute to vascular maturation. Interactions between GBM cells and pericytes facilitate vessel co‐option and tumor cell migration by altering cellular contractility and promoting aberrant vascular remodeling.	A perfusable, patient‐derived vascularized GBM model: co‐printing patient‐derived GBM cells, astrocytes, and microglia. After removal of the Pluronic, pericytes and HUVECs were seeded.	[168, 169, 170, 171, 172]
	Brain microvascular endothelial cells (BMECs)	GBM is its abundant and aberrant vas‐culture. Some proangiogenic factors like VEGF are upregulated. This leads to the migration and proliferation of ECs, which is a key event in the angiogenic cascade. Vascular growth is essential for GBM invasion and progression.	A vascularized microfluidic model called GlioFlow3D: a sacrificial Pluronic template, lined with human BMECs, and interfaced with astrocytes and U87 GBM cells embedded in a fibrinogen hydrogel.	[173, 174]
	CAFs	These cells have been shown to have pro‐tumoral effects through interactions with GBM stem cells, leading to M2 macrophage polarization. The abundance of CAFs in GBM is closely correlated with higher tumor grade, poor clinical outcomes, and increased ECM remodeling activity. CAFs secrete CCL2, which enhances temozolomide resistance in GBM cells through CCL2‐CCR2‐mediated signaling.	Vascularized GBM spheroid model: fibroblasts and HUVECs were embedded in a gelatine/alginate/fibrinogen bioink. U87 cell spheroids were then seeded into the vascularized layer.	[175, 176, 177]
	Neurons	Glioma cells tend to grow in proximity to neurons, playing a pivotal role in glioma development. Neurons were demonstrated to stimulate glioma growth by secreting neuroligin‐3, which enhances glioma cell depolarization and proliferation.	G/N models: an outer hemispherical shell with alginate and neurons and an inner hemisphere with alginate and GL261 cells.	[178, 179, 180, 181]
Breast cancer	CAFs	CAFs in conjunction with inflammation result in tissue fibrosis [24]. CAFs secrete growth factors promoting angiogenesis, immunosuppression, EMT, as well as tumor cell proliferation, survival, invasion, and metastasis. CAF signaling pathways such as oxidative stress, autophagy, and glycolysis promote the generation of a microenvironment suitable for tumor cell growth and expansion.	Chitosan‐alginate blends: co‐culture CAFs, BC cells, and T‐lymphocytes.	[182, 183, 184, 185, 186, 187]
	CAAs	Adipocytes are an abundant cell type found within the mammary gland. CAAs secrete various cytokines and adipokines that promote tumor progression. This dysregulated secretion, combined with free fatty acids and MMPs, results in the recruitment of immune cells to the TME, creating an environment akin to chronic inflammation, promoting EMT and an invasive tumor phenotype.	Tumor spheroids (using human cell lines, HCC1806 and MDA‐MB‐231) were seeded in microwells of soft or stiff GelMA gels, in which preadipocytes were encapsulated.	[188, 189, 190, 191, 192, 193]
	ECs	Cells stimulate angiogenesis by secreting VEGF and fibroblast growth factor 2. Due to the rapid growth of vasculature and a lack of some cytokines, they help the invasive tumor cells intravasate through the vessels. Along with additional endothelial–tumor cell interactions further accelerate tumor growth and metastasis.	Pre‐neoplastic (MCF10AT1‐EIII8) breast epithelial cells were embedded in Matrigel in the presence of HUVECs and normal fibroblasts or CAFs.	[194, 195, 196]
OS	MSCs	Bone marrow‐derived MSCs are a key component of the OS TME, exhibiting a pronounced affinity for OS cells. MSCs generate osteoblasts and support hematopoietic stem and progenitor cells, which give rise to immune cells and osteoclasts. MSCs confer drug resistance and foster the stemness of CSCs by membrane crosstalk via extracellular vesicle secretion, metabolic reprogramming of tumor cells, and immune escape. The growth‐promoting effect of IL‐6 and VEGF secreted by MSCs when MSCs are co‐cultured with OS cells.	A 3D bone‐mimetic model of OS (mOS‐3D) by co‐culturing human OS cells and MSCs within a hydroxyapatite–collagen scaffold.	[197, 198, 199]
	CAFs	Upon interaction, bone marrow MSCs differentiate into CAFs and secrete cytokines such as IL‐6, IL‐8, and MCP‐1 within the TME. These factors collectively promote increased OS invasiveness, motility, and trans endothelial migration.		
	Osteoblasts	OS cells secrete parathyroid hormone‐related protein, IL‐6, and IL‐11, which activate osteoblasts. In turn, osteoblasts produce receptor activator of nuclear factor‐κB ligand, thereby promoting osteoclast activation and sustaining bone resorption and tumor invasion. The activated osteoblasts lay down immature woven bone, producing ectopic osteoid lesions which may impinge on surrounding nerve structures	Methacryloyl plateletlysates (PLMA)‐based hydrogels: OS spheroids co‐cultured with MSCs and osteoblasts.	[200]
	Osteoclasts	Osteoclast activity releases ECM growth factors, further stimulating tumor growth and sustaining the destructive cycle. The substantial osteolysis exhibited by some OS subtypes is the direct result of increased osteoclastic activity.		
Lung cancer	CAFs	Correlation between TGF ‐ β secretion from CAFs and tumor heterogeneity in lung adenocarcinomas. TGF ‐ β stimulated lung adenocarcinoma tumor plasticity into acinar type. Hence, TGF ‐ β mediates a certain role in cell‐to‐cell crosstalk between tumor and TME. It is also involved in ECM changes during progression and initiates bone metastasis.		
	Pericytes	Pericytes play an important role in tumor progression through the formation of tumor stroma, immune cells regulation, tumor angiogenesis, and neovascularization development, and engagement in defeating the brain–blood barrier through lung cancer brain metastasis formation.		
	MSCs	MSCs produce vimentin, MMPs, N‐cadherin, fibronectin, integrins, and smooth muscle actin, which form a foundation of the tumor ECM. MSCs also express chemokines involved in the recruitment of immune cells and stromal cells toward increasing tumor cell mobility and overcoming the venous barrier during neovascularization across EMT.	3D microspheres: A549, fibroblasts, and bone marrow‐derived MSCs on HA microparticles.	
PDAC	CAFs	CAFs represent the principal stromal cell population, secreting ECM proteins such as collagen, fibronectin, and HA. These cells facilitate tumor progression through cancer cell proliferation, induced inflammation, ECM remodeling, and immune suppression through cytokine signaling networks such as TGF‐β, IL‐6, GM‐CSF, and ROS.	A PDAC bio‐printed model: combining PDAC cells (Panc‐1) with CAFs encapsulated in a sodium alginate and gelatin‐based hydrogel.	[201, 202]
	PSCs	PSCs contributed to ECM deposition by showing increased levels of fibronectin and collagen I, and were activated as shown by increased α‐SMA expression.	PDAC‐on‐a‐chip platform: pancreatic tumor cells and PSCs embedded in a 3D collagen matrix.	[159]

These co‐culture strategies collectively emphasize that stromal cells actively influence tumor phenotype and therapy response. However, most systems still rely on simplified cell compositions and static setups. To capture the evolving crosstalk seen in vivo, future models should incorporate patient‐derived immune and stromal cells with adaptive feedback mechanisms and longitudinal monitoring.

### 4.4 Engineering Specific Pathophysiological Processes

#### 4.4.1 Angiogenesis and Vascularization

Early tumor growth occurs during an avascular phase, during which the lesion remains nonaggressive and relies on passive diffusion from adjacent tissues to obtain oxygen and nutrients. As tumors expand, they establish a hostile microenvironment characterized by hypoxia, ischemia, acidosis, and elevated interstitial pressure. These conditions stimulate the secretion of growth factors and cytokines within the TME, thereby initiating angiogenesis and lymphangiogenesis. In vivo, tumor angiogenesis proceeds through multiple mechanisms, including sprouting angiogenesis, intussusception, vasculogenesis, vessel co‐option, and vasculogenic mimicry (VM), as comprehensively summarized by Liu and colleagues [203].

VM describes the ability of tumor cells to elongate and organize into vessel‐like channels that connect with the host vasculature, facilitating the delivery of oxygen, nutrients, and blood. This process is thought to be driven by hypoxia‐induced secretion of MMPs and periodic acid Schiff positive matrix components [204]. Reproducing VM in 3D in vitro models requires careful consideration of multiple microenvironmental cues. Notably, serum, which is routinely added to cancer cell cultures, can modulate VM by activating signaling pathways and altering the expression of key regulators such as vascular endothelial cadherin (VE‐cadherin), twist, MMP, aquaporin‐1, and LAMC2 [30, 205, 206, 207]. For example, prostate cancer cell lines cultured in serum‐containing, Matrigel‐polymerized 24‐well plates exhibited serum‐driven Sp1 upregulation, which increased nuclear Twist levels, enhanced VE‐cadherin transcription, and activated the downstream AKT‐MMP‐2‐LAMC2 axis. Together, these events promoted ECM remodeling and channel formation characteristic of VM [208]. In addition to serum stimulation, hypoxia alone can act as an independent inducer of VM‐supportive phenotypes. Vera et al. exposed SKOV3 ovarian cancer cells to 48 h of hypoxia and subsequently seeded them onto Matrigel under serum‐free conditions. Within 12 h, the cells formed early 3D networks resembling VM structures [209].

Given the central role of the tumor‐vascular microenvironment in tumor growth, dissemination, and therapeutic response, the development of vascularized in vitro models is crucial for accurately replicating cancer biology. One widely adopted strategy involves embedding ECs within 3D ECM scaffolds and exploiting their intrinsic ability to form lumenized, interconnected vascular networks. These endothelial linings actively regulate tumor cell intravasation, vascular remodeling, and the transport of fluids and soluble factors [203, 210, 211, 212, 213, 214, 215]. For example, HUVECs and dermal fibroblasts were co‐embedded in a low‐dose 0.5% adipose dECM supplemented with fibrin inside a 3D‐printed microfluidic chip and cultured on a gentle rocker. Under these conditions, ECs self‐assembled into stable, lumenized networks with increased vessel diameter. The adipose dECM enhanced angiogenic programs through activation of PI3K‐ and JAK‐mediated signaling pathways. When colon cells were incorporated into this system, perfused microvessels significantly promoted tumor growth, demonstrating functional tumor‐vasculature crosstalk and establishing a robust platform for mechanistic studies and drug testing [213].

Despite these advances, vascularization patterns vary substantially across tissues and organs, and many existing models remain limited in their ability to recapitulate in vivo vascular networks with respect to size, geometry, and spatial distribution [216]. To achieve more precise control over angiogenesis in vitro, several engineering strategies have been developed. Microneedles have been used to create predefined microchannels within ECM scaffolds to guide EC seeding [215]. Microfluidic devices incorporating such endothelial channels enhance tumor spheroid migration, invasion, and angiogenesis [217]. Microneedle templating enables the rapid fabrication of perfusable, endothelialized microvessels with defined diameters and controllable hemodynamic conditions, while requiring relatively few cells and reagents. Even so, withdrawing the needle after hydrogel polymerization largely confines the resulting vessels to straight channels, thereby limiting the reconstruction of branched capillary networks. For constructing tumor‐vascular interfaces, ring magnets placed above and below culture modules enable rapid, self‐aligned integration of endothelial‐channel Transwell inserts with PDMS‐hydrogel microwell spheroid plates. Magnetic fixation acts as a noninvasive clamp during fabrication and operation, stabilizing components during sacrificial‐layer removal and culture, while facilitating EC attachment to tumor spheroids [218]. However, these approaches often struggle to reproduce the complexity of microvasculature below 200 µm in diameter, which is widely distributed throughout the body. To address this limitation, Salvadori et al. employed femtosecond near‐infrared laser photoablation to directly generate cylindrical microchannels within standard PDMS microfluidic chips, achieving channel diameters of 50–80 µm and interchannel spacing of ≤100–200 µm. Following HUVEC seeding, the resulting media channels exhibited intact endothelial junctions and preserved endothelial identity, highlighting the platform's potential for vascularized tumor‐on‐chip models in contemporary oncology and immunotherapy research [219]. Femtosecond laser photoablation enables the rapid fabrication of microvascular channels with high structural reproducibility. Conversely, its broad application is constrained by the need for specialized laser equipment and by the difficulty of achieving stable endothelialization in channels below approximately 80 µm.

Vascularized models thus bridge a gap between avascular spheroids and in vivo tumors, offering a platform to study perfusion, drug delivery, and metastatic trafficking. Nevertheless, achieving physiological barrier function, hierarchical branching, and stable perfusion remains technically demanding. Convergence of bioprinting, microfluidics, and organ‐specific ECs will advance the development of vascularized tumor models.

#### 4.4.2 Cancer Cell Stemness

CSCs were first identified by Bonnet in nonobese diabetic/severe combined immunodeficiency mice and have since been validated across a wide range of solid tumors [220]. Owing to their unique biological properties, CSCs play pivotal roles in tumor initiation, progression, and therapeutic resistance. First, CSCs exhibit relative immortality through self‐renewal, sustaining the long‐term maintenance of the bulk tumor cell population. Second, their capacity for multidirectional differentiation contributes to tumor heterogeneity and phenotypic diversity [221]. Moreover, CSCs promote invasion and metastasis by inducing immunosuppressive, metastasis‐supportive microenvironments and by transitioning into metastasis‐initiating cells [221].

Conventional in vitro enrichment of CSCs typically relies on suspension culture systems, such as spheroid formation assays, which are time‐consuming and often inefficient [222, 223, 224]. With the development of 3D scaffold‐based hydrogels, numerous biomaterials have been identified that selectively induce anoikis in non‐stem cancer cells, thereby enriching CSC populations [96, 152, 225]. For example, Rao et al. cultured prostate cancer cells in CSC‐promoting medium and encapsulated them within an alginate‐based hydrogel to generate core–shell microcapsules. This system significantly shortened the time required to form CSC‐containing spheroids and increased the expression of stemness markers and dye‐exclusion capacity [226]. Such models have enabled in‐depth exploration of the genetic and epigenetic characteristics of CSCs [227].

The concept of the CSC niche has further highlighted the critical roles of ECM biophysical and biochemical properties in maintaining stemness [228]. Consequently, extensive efforts have focused on elucidating how ECM characteristics, including matrix stiffness [229, 230], viscoelasticity [231], and molecular and cellular components [142], influence the underlying mechanisms. At the same time, there is a pressing need for standardized, cost‐effective, and scalable culture platforms that can efficiently enrich and maintain CSCs across multiple models [232, 233, 234, 235].

3D bioprinting offers a promising solution to this challenge by enabling the precise reconstruction of CSC niche architecture. In one representative study, researchers employed 3D bioprinting to engineer an in vitro niche for breast CSCs using a tri‐compartmental tumor–stroma model. The system utilized a dual‐bioink strategy consisting of (i) a tumor bioink containing TNBC CSCs derived from MDA‐MB‐231 cells suspended in porcine breast tissue‐derived dECM, and (ii) a stromal bioink composed of human breast fibroblasts embedded in the same dECM. To overcome the low viscosity of dECM, the authors implemented a support‐bath bioprinting approach, in which the tumor bioink was extruded as a multilayered circular core, approximately 1 mm in diameter, directly into a surrounding stromal bioink reservoir. The unique viscoelastic properties of dECM enabled precise spatial patterning, preserving tumor core architecture while permitting natural cell–ECM interactions. Post‐printing incubation at 37°C induced simultaneous crosslinking of both compartments, generating a biologically relevant 3D microenvironment in which CSC clusters were concentrically surrounded by stromal cells, closely mimicking the native organization of breast carcinoma. This bioprinted model successfully maintained CSC stemness, upregulated canonical stem cell markers, and recapitulated functional CSC traits, including invasive behavior and drug resistance, thereby validating its utility for modeling critical aspects of the CSC niche [236].

3D biomimetic scaffolds therefore provide a physiologically relevant context to study CSC regulation and targetability. However, CSC enrichment remains variable across protocols, and niche‐mediated resistance mechanisms are still poorly understood. Standardized, mechanically tunable platforms combined with lineage tracing and single‐cell analytics will be essential to unravel the dynamic interplay between CSCs and their microenvironment.

#### 4.4.3 Invasion and Metastasis

Metastasis is a multistep biological program initiated when a subset of tumor cells undergoes EMT and acquires an invasive, motile phenotype. These cells remodel and breach the basement membrane, intravasate into blood or lymphatic vessels, and disseminate as circulating tumor cells (CTCs). Platelet cloaking of CTCs promotes immune evasion and facilitates vascular arrest and extravasation. At distant sites, disseminated cells adapt to the local niche, re‐enter proliferative programs, and ultimately colonize to form clinically detectable metastases [237, 238]. To dissect the mechanisms governing individual steps of metastasis, investigators increasingly rely on 3D scaffold‐based models that recapitulate essential features of the TME (Figure 3).

**FIGURE 3:**
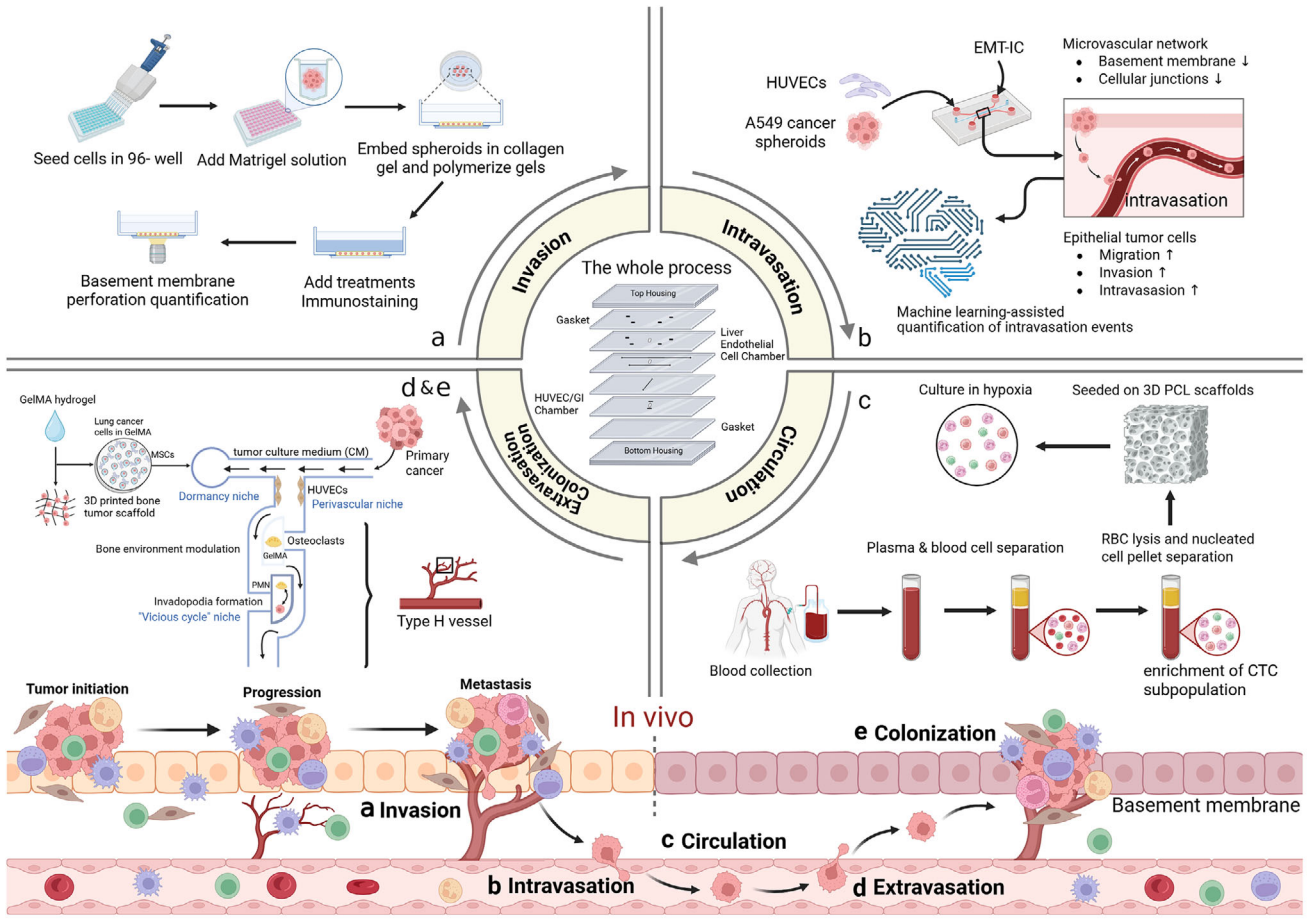
Tumor metastasis in vivo typically proceeds through five sequential steps: invasion, intravasation, circulation, extravasation, and colonization. Advances in 3D biomimetic scaffold technologies now enable these key stages to be reconstructed and systematically studied in vitro. (a) Invasion: The Transwell/Matrigel invasion assay represents a classical 3D platform for assessing the ability of tumor cells to penetrate the basement membrane; (b) Intravasation: Chip‐based tumor models that integrate extracellular matrix cues with functional, perfusable microvascular networks permit real‐time visualization and quantitative analysis of EMT‐driven intravasation of cancer cells into the vasculature; (c) Circulation: PCL scaffolds can be used to enrich and CTC subpopulations, thereby mimicking the survival conditions encountered in the bloodstream and providing a platform for downstream phenotypic and functional analyses of CTCs; (d & e) Extravasation and colonization: The combination of GelMA hydrogels with bone microenvironment‐associated cells enables reconstruction of the perivascular or dormancy niche characteristic of bone metastasis. This system facilitates investigation of cancer cell colonization following extravasation. CTC, circulating tumor cells; HUVEC, human umbilical vein endothelial cell; EMT, epithelial–mesenchymal transition.

Local invasion represents the first step of the metastatic cascade, during which tumor cells acquire motile phenotypes, remodel the surrounding ECM, and breach the basement membrane. A common approach involves tuning ECM composition and mechanical properties, and quantifying the resulting invasive behaviors [57, 81, 152, 239, 240, 241]. Zhao et al. crosslinked HA of varying molecular weights with alginate to culture BC cells and demonstrated that low‐molecular‐weight HA35 robustly enhanced invasion of 4T1 and SKBR3 cells via activation of a CD44‐Twist signaling axis [242]. Similarly, type I collagen‐based matrices have been shown to reprogram tumor cell motility through transcriptional and epigenetic mechanisms across multiple cancer types. In a senescent niche model, embedding BRAFV600E melanoma cells within senescent collagen I fibrils increased invasive behavior and resistance to vemurafenib through elevated YAP signaling [134, 239]. These matrix models facilitate reproducible manipulation and mechanistic interrogation of tumor–matrix interactions, although the absence of stromal, vascular, and immune components limits faithful recapitulation of invasion in vivo. The Transwell/Matrigel invasion assay remains a standard 3D platform for assessing basement membrane traversal, enabling quantification of protease‐dependent degradation and cell migration [243, 244]. Building upon this assay, advanced bioengineered systems offer precise control over geometry and material properties, allowing researchers to disentangle ECM biochemical cues from biophysical effects. For example, Nazari et al. encapsulated tumor spheroids within a basement membrane extract and embedded them in a surrounding collagen IV stromal gel. Live‐cell imaging revealed nucleation and expansion of perforations in the membrane layer, with invasion supported by protrusion‐mediated integrin adhesions along basement membrane lacunae [245, 246]. Complementarily, Khoo et al. embedded MDA‐MB‐231 cells in composite hydrogels with independently tunable stiffness and pore or fiber density, demonstrating that matrix mechanics and microarchitecture independently and synergistically regulate invasive behavior [241].

Intravasation requires coordinated interactions with stromal cells and a functional endothelial barrier, features that conventional invasion assays fail to capture [247]. To address this gap, tumor‐on‐a‐chip (ToC) platforms have been developed to integrate stromal cues with functional and perfusable microvascular networks. In one such human microvasculature‐on‐a‐chip model, endothelial microvessels were generated by embedding HUVECs within a fibrin matrix inside a three‐channel microfluidic device. Vascular identity and barrier integrity were validated using permeability assays. When lung cancer spheroids embedded in the MVN‐containing gel were exposed to an EMT‐inducing cocktail, tumor cells breached the endothelial barrier and intravasated into perfused microvessels. This system enables real‐time visualization and quantification of the rate‐limiting step of metastasis, providing physiological relevance beyond that of conventional invasion assays [248].

Because CTCs circulate in the bloodstream, they can be accessed through routine blood sampling, positioning them as valuable resources for metastasis research and as clinically actionable biomarkers [50]. However, conventional CTC detection methods are often labor‐intensive and exhibit limited sensitivity and specificity. To overcome the low yield and bias associated with marker‐dependent approaches, researchers cultured red blood cell‐lysed nucleated cells from single 10‐mL blood samples of treatment‐naïve metastatic BC patients directly within a PCL scaffold under mild hypoxic conditions. The salt‐leached PCL scaffold recapitulated tumor‐like stiffness and supported deposition of native ECM, thereby preserving cell–cell and cell–matrix interactions absent in 2D cultures. Across 16 patient samples, this platform successfully recovered and expanded CK^+^/CD45^−^ CTCs in 12 cases without prior antigen‐based enrichment, enabling downstream phenotypic analyses. By avoiding fixation and selection markers, this approach offers a practical and scalable strategy for studying CTC biology and therapeutic responses with improved fidelity and throughput [50].

Metastatic dissemination culminates when CTCs extravasate from capillaries, occupy perivascular niches in the target organ, and adapt to local cues to establish overt lesions. To interrogate these late‐stage events, researchers have engineered distal organ‐mimetic platforms seeded with primary tumor‐derived cells. These systems recapitulate organ‐specific vascular arrest, transendothelial migration, and the dormancy‐to‐outgrowth transition, enabling controlled studies of colonization in a physiologically relevant context [249, 250, 251, 252, 253]. For example, Ji et al. combined 3D printing with a bone‐on‐a‐chip device to reconstruct pre‐metastatic bone niches. Bone‐resident cells were encapsulated in photocrosslinked GelMA hydrogels to mimic the architecture of the marrow. At the same time, adjacent microchannels were patterned to represent type H vasculature and lined with HUVECs to establish a perivascular compartment. Upon introduction of A549 lung cancer cells, the platform enabled direct visualization and quantification of invadopodia formation through cortactin and Tks5 activation, revealing paracrine crosstalk‐dependent switches between dormancy and reactivation [254]. This platform offers a physiologically relevant framework for interrogating bone metastatic dormancy and reactivation, yet its translational value remains constrained by incomplete vascular and immune recapitulation and the absence of in vivo validation.

Because metastasis in vivo is a dynamic and continuous process, reductionist in vitro models that isolate individual steps rarely capture the coordinated molecular programs and multicellular interactions that drive dissemination. Accordingly, the field is shifting toward platforms that integrate multiple rate‐limiting stages or even reconstruct the whole sequence from local invasion through intravasation, circulation, extravasation, and colonization. By modeling these events within a single, coherent framework, such systems enable a mechanistically rigorous, system‐level interrogation of metastasis and provide more predictive testbeds for developing and prioritizing therapies that interrupt dissemination and outgrowth [255].

These integrated models provide a comprehensive framework for investigating the metastatic cascade. Yet, capturing the full cascade from local invasion to colonization in a single platform remains a formidable challenge. Future efforts should focus on coupling multi‐organ microphysiological systems with CTC analysis to model patient‐specific metastatic trajectories and niche‐dependent outgrowth.

## 5 Applications in Cancer Research and Drug Development

### 5.1 Drug Screening and Discovery

The primary goal of preclinical drug screening is to predict patient responses to standard therapies, a task increasingly enabled by biomimetic in vitro tumor models (Figure 4). By precisely tuning the composition, cellular ratios, and physical properties of 3D scaffolds, researchers can reconstruct the TME and emulate distinct stages of tumorigenesis, including initiation, expansion, invasion, and metastasis. Compared with conventional 2D monolayer cultures, 3D systems more closely approximate in vivo constraints, support long‐term tumor cell growth, and often induce higher levels of drug tolerance. As a result, they provide more discriminative and clinically relevant assessments of therapeutic efficacy [43, 256, 257, 258, 259, 260, 261]. For example, ER^+^/HER2^+^ BC cells cultured in Matrigel‐based 3D systems exhibit reduced sensitivity to endocrine therapies and trastuzumab relative to monolayer cultures [256]. Similarly, collagen‐based scaffolds have been used to model glioma behavior, revealing enhanced chemoresistance associated with an increased proportion of glioma stem cells [257]. Furthermore, engineered 3D scaffold models are frequently more scalable and experimentally tractable while retaining essential microenvironmental cues, thereby supporting near‐term translational applications [262]. In a bioreactor‐based 3D culture system, freshly resected colorectal cancer fragments are maintained ex vivo between two collagen layers in a “sandwich” configuration, preserving tissue architecture and proliferative cell density for up to three days and enabling short‐term prediction of chemotherapy response [262]. Collectively, 3D models offer substantial promise for drug discovery and testing by recapitulating native tissue architecture and revealing pathological alterations throughout tumor progression [152, 263, 264, 265]. Phytochemicals have been extensively investigated for their high safety profile and multifaceted antitumor potential, including modulation of immune function, inhibition of tumor growth, and prevention of tumor recurrence and metastasis. Zhu et al. employed sodium alginate/gelatin microbeads to demonstrate the cytotoxicity of crocin toward MCF‐7 cells in vitro [266]. Han et al. further showed that resveratrol suppresses VM in prostate cancer cells cultured in Matrigel by disrupting the EphA2/twist‐VE‐cadherin/AKT signaling axis [267]. Additionally, isolinderalactone was validated in a microfluidic chip model to critically inhibit the survival, proliferation, and migratory capacity of GBM cells [265].

**FIGURE 4:**
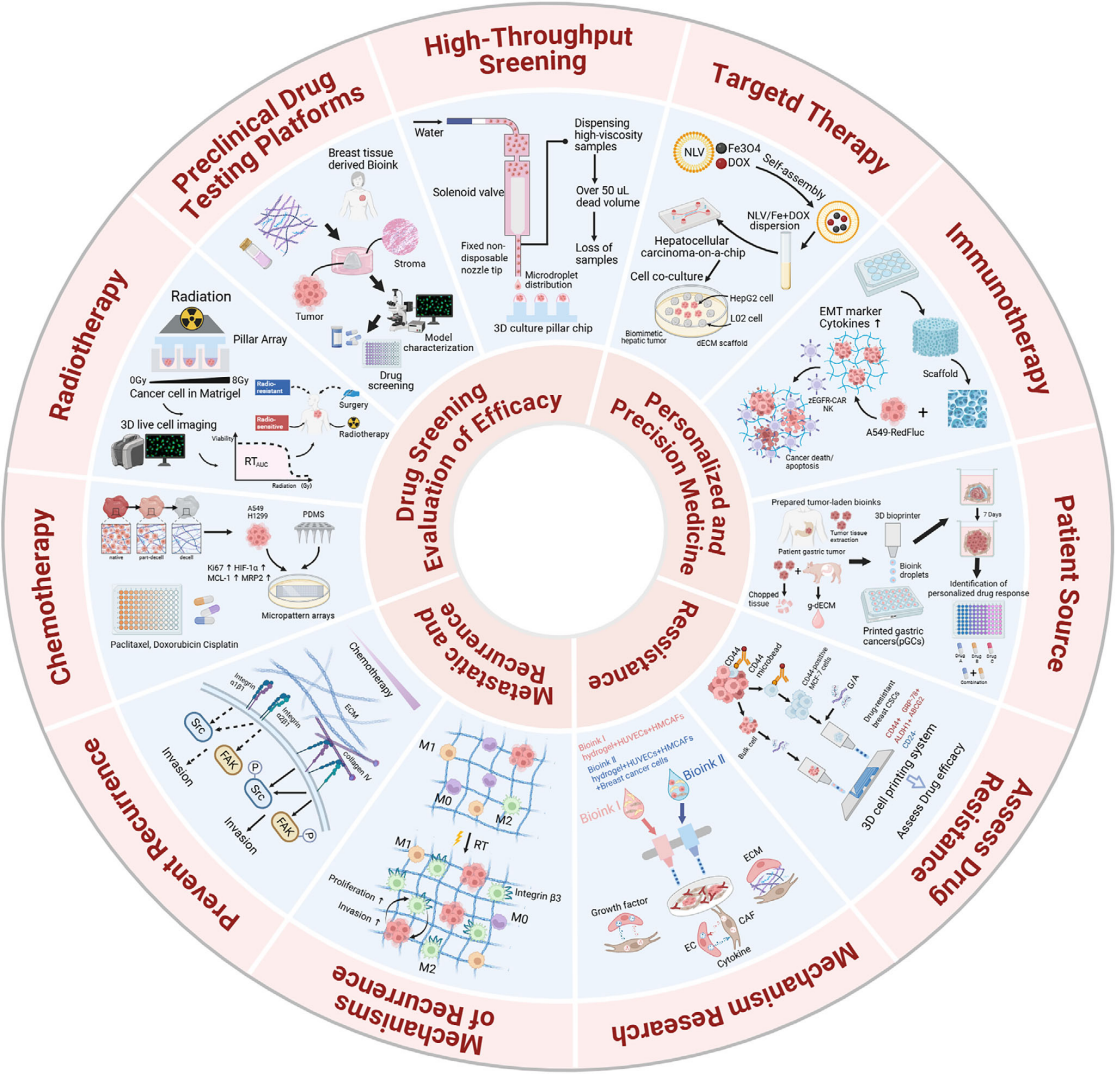
Schematic diagram of the major applications of 3D biomimetic scaffolds in cancer research, highlighting their increasingly important roles in drug screening, precision therapy, and the investigation of mechanisms underlying drug resistance.

In recent years, preclinical drug testing platforms have become increasingly sophisticated. A wide range of biomaterials and fabrication strategies have been employed to mimic the TME, thereby reproducing tumor ecological niches closely and more faithfully. By establishing controlled drug concentration gradients within these systems, it is possible to predict clinical responses to standard therapies better. For example, HepG2 cells maintained for 21 days in a decellularized liver matrix (DLM)‐GelMA hydrogel displayed linear, dose‐dependent responses to acetaminophen and sorafenib toxicity [268]. In another study, human BC cell lines MDA‐MB‐231 and MCF‐7 were embedded in a HAMA‐modified decellularized porcine breast matrix and co‐cultured with stromal cells to form 3D core–shell constructs via bioprinting. Upon exposure to increasing concentrations of doxorubicin and paclitaxel, MCF‐7 models exhibited dose‐dependent cytotoxicity, whereas MDA‐MB‐231 models showed no clear trend, highlighting subtype‐specific resistance mechanisms [269]. To enable precise monitoring of drug distribution across different spatial compartments, surface‐enhanced Raman scattering sensors were integrated into the core–shell BC model. These sensors revealed differential consumption of the antitumor drug 6‐thioguanine between the tumor core and stromal regions, thereby providing the analytical sensitivity required to evaluate compartment‐specific cellular responses to chemotherapy [270]. Given the complexity of in vivo pharmacodynamics and pharmacokinetics, it is crucial to model drug diffusion from vascular networks into the TME rather than relying solely on direct immersion. This can be achieved by precisely controlling the spatial arrangement of vascular and tumor tissues. In this context, ToC systems with embedded microchannels offer distinct advantages for simulating drug delivery. For example, Park et al. developed a 3D vascularized lung cancer‐on‐a‐chip featuring two cylindrical macrochannels within hybrid hydrogels to mimic perfusable large‐sized vessels. During drug testing, molecules were perfused through the microfluidic channels and subsequently diffused into the surrounding ECM, establishing a physiologically relevant drug gradient within the scaffold. Compared with non‐vascularized hydrogels, this model exhibited reduced doxorubicin‐induced apoptosis alongside sustained caspase‐3 activation, underscoring the importance of vascular context in evaluating dose‐dependent treatment effects [271].

High‐throughput drug screening further requires standardized control over the size, morphology, and spatial arrangement of 3D tumor models. Such systems are commonly fabricated on 96‐ or 384‐well plates or 330‐ and 532‐micropillar/well chips. Considerable research efforts have been devoted to achieving precise and reproducible control over the morphology and dimensions of these models. For instance, Zhu et al. cultured lung cancer cell lines on dECM‐coated micropatterns of varying diameters using PDMS seals to evaluate the cytotoxicity of paclitaxel, doxorubicin, and cisplatin. They observed that cells cultured on micropatterns with diameters of 100–150 µm maintained structural integrity over 3 days without signs of aggregation or overcrowding, while also exhibiting a chemoresistant phenotype [272]. Bioprinting technologies offer additional advantages by enabling automated, high‐precision deposition of cell‐laden hydrogels. Extensive work has been conducted to optimize 3D bioprinted constructs by adjusting parameters such as nozzle diameter, printing speed, and pressure, in addition to modifying bioink composition. Compared to manually pipetted scaffolds, the bioprinting approach significantly reduces processing time per plate and ensures more consistent volumetric distribution of hydrogels for each printed 3D microtumor [273, 274]. In a recent advancement, Jeong et al. developed the ASFA SPOTTER V6, a micro‐solenoid valve‐based bioprinting system capable of distributing highly viscous biosamples rapidly and in minute volumes without compromising cell viability, thereby overcoming limitations associated with inkjet‐ and acoustic‐based methods [275]. Droplet microfluidics has also emerged as a valuable tool for high‐throughput anticancer drug screening, owing to its ability to encapsulate cells within hydrogel microbeads at high droplet generation rates on the order of 10^4^ droplets per minute while maintaining highly uniform droplet size [276].

For combinatorial drug screening, 3D ECM microsystems that integrate in silico diffusion modeling with microfluidic experimentation allow parallel and spatially controlled testing of multidrug regimens across graded concentrations. By positioning tumor fragments adjacent to diffusion‐aligned microchannels and applying the combination index theory, these platforms enable the precise prediction and experimental validation of synergistic drug interactions [277].

3D models thus offer a more predictive bridge between traditional screening and clinical outcomes, especially for drugs whose efficacy is microenvironment‐dependent. However, throughput and scalability still lag behind 2D systems, and validation against patient response data remains limited. Harmonizing assay readouts, automating image analysis, and establishing biobanks of patient‐derived scaffold models will accelerate their adoption in preclinical pipelines.

### 5.2 Molecular Targeted Therapy

Systemic chemotherapy is often limited by poor selectivity, short intratumoral residence times, and subtherapeutic exposure at disease sites. To overcome these limitations, contemporary targeting paradigms focus on both tumor‐intrinsic vulnerabilities and microenvironmental reprogramming [278, 279, 280]. Guided by comprehensive genomic and transcriptomic profiling, a range of small‐molecule inhibitors and therapeutic antibodies have been developed to selectively disrupt oncogenic drivers and survival pathways in cancer cells. In parallel, the TME has become an equally important therapeutic target, with strategies aimed at normalizing tumor vasculature and remodeling stroma to curb progression and delay therapeutic resistance [280].

Tumor‐intrinsic molecular targeting exploits cancer‐specific molecular alterations to selectively inhibit oncogenic drivers and dysregulated signaling pathways that sustain malignant cell proliferation and survival [281]. Nonetheless, the therapeutic efficacy and durability are shaped by genetic and epigenetic heterogeneity, target alterations, and microenvironmental barriers that are poorly reproduced in conventional 2D cultures [282]. Biomimetic 3D tumor models therefore provide a more physiologically relevant framework for evaluating established targeted therapies. Bahraminasab and colleagues optimized a gelatin–sodium alginate hydrogel system for evaluating CD73 inhibition in GBM. The formulation containing 5% gelatin and 5% sodium alginate showed the most favorable rheological and cytocompatibility profiles, providing a stable matrix that supported GBM cell adhesion and aggregation. Within this optimized matrix, CD73 inhibition reduced tumor‐cell proliferation and suppressed VEGF and HIF‐1α expression [283]. Extending this approach to combination testing, Omran et al. established a Matrigel‐based 3D uterine sarcoma model using MES‐SA cells to assess tazemetostat and entinostat, either alone or in combination. Dual inhibition produced greater antitumor activity than either monotherapy, evidenced by reduced proliferation, enhanced apoptosis, and cell‐cycle arrest [284]. Despite these encouraging findings, the reliance on monoculture‐based systems and the lack of in vivo validation limit the ability of these models to reliably predict clinical responses.

Tumor progression is accompanied by dynamic remodeling of the ECM, driven by reciprocal interactions between cancer cells and stromal components. Due to the characterized biochemical and physical cues, the remodeled ECM has emerged as actionable targets for anticancer intervention [285]. Capitalizing on this, the strategic design of nanocarriers that target tumor‐specific ECM components presents a promising theoretical framework for enhancing drug accumulation within the TME. Integrins, heterodimeric transmembrane receptors composed of α and β subunits, serve as critical ECM‐cytoskeleton linkers and signaling hubs. Aberrant integrin expression on tumor and stromal cells has been documented across multiple malignancies, where they profoundly influence the tumor niche by orchestrating multiple downstream signaling pathways [133]. In a 3D collagen gel culture model mimicking the bladder TME, application of the integrin α2β1 ligand peptide TFA effectively reversed doxorubicin resistance in bladder cancer cells. This reversal was achieved by disrupting the collagen–integrin interaction, thereby inhibiting the subsequent activation of the integrin β1/AKT signaling axis that confers chemoresistance [286]. In another study, MacDonald and colleagues adopted a broader TME‐directed strategy by establishing an invasive 3D pancreatic cancer co‐culture model incorporating both tumor cells and PSCs. Necuparanib suppressed tumor invasion by disrupting tumor–stromal signaling and reducing MMP activity, with corresponding decreases in MMP1 and increases in TIMP3. These effects were supported in mouse models and treated patient plasma samples. Collectively, this study highlights the value of integrating 3D coculture, animal models, and clinical biospecimens when evaluating targeted therapies [287].

The utilization of HUVEC‐based vascular‐on‐a‐chip models enables researchers to monitor in real time the inhibitory effects of antiangiogenic drugs on endothelial sprouting [218, 288]. Coupled with quantitative diffusion analysis to assess alterations in vascular barrier function, this platform provides critical insights for guiding clinical drug application and facilitating efficient drug screening. Gu and colleagues established a perfusable and leak‐free humanized vascular model in vitro by fabricating EC‐laden tubular constructs via coaxial bioprinting, which were mechanically supported by an integrated PCL stent. The platform enabled the dynamic evaluation of bevacizumab efficacy through real‐time monitoring facilitated by fluorescence microscopy, confocal microscopy, and a live‐cell incubation system, demonstrating its critical value in antiangiogenic drug screening [288].

### 5.3 Nanomedicine

Nanotechnology and advanced biomaterials provide versatile platforms for precision cancer therapy by enabling controlled drug delivery, remodeling of the TME, and direct targeting of malignant cells. Rationally engineered nanocarriers protect therapeutic payloads from premature degradation, improve their solubility, prolong circulation time, and facilitate sustained drug release [289]. To reliably evaluate these sophisticated functionalities, various ToC platforms have been developed to simulate the dynamic pharmacokinetic profiles of multifunctional nanocarriers under physiologically relevant conditions. These microfluidic systems accurately recapitulate key in vivo features, enabling real‐time assessment of nanodrug accumulation, penetration, and efficacy within tumor tissues [213, 214, 215]. Liu et al. developed a controllable, high‐throughput screening platform for nanocarrier evaluation in neuroblastoma by integrating an automated concentration gradient generator (CGG) chip with a 3D vascularized co‐culture model [290]. This system generated uniform concentration gradients of nanoparticles, which were delivered via perfusion through connected microchannels, effectively mimicking systemic drug administration. Notably, the application of parallelized microfluidic architecture enabled simultaneous processing across multiple culture channels, demonstrating its capacity for high‐throughput nanomedicine screening [290].

Numerous nanoparticles have increasingly been explored as multifunctional therapeutic platforms capable of overcoming barriers within the TME [291]. The deposition of fibrillar collagen increases the density and stiffness of the ECM, which consequently acts as a major physical barrier to drug penetration. The integrated photothermal therapy was applied to overcome this barrier. Sae‐Be et al. designed a sequential nanotheranostic platform, wherein aminated hollow mesoporous silica nanoparticles loaded with doxorubicin were surface‐capped with graphene quantum dots. Upon near‐infrared laser irradiation, these nanoparticles enable localized hyperthermia, effectively degrading the surrounding collagen fibers. The utilization of 3D in vitro models facilitates rapid and convenient qualitative analysis of collagen within the TME. Specifically, by combining immunofluorescence staining with high‐content imaging analysis, this platform allows for the quantitative assessment of type I collagen depletion, thereby providing a direct measure of the nanoparticle's ability to disrupt the stromal barrier and enhance drug penetration [292].

Despite advances in nanocarrier engineering, achieving selective recognition of malignant tissues remains challenging due to intratumoral heterogeneity and complex microenvironments. Passive tumor accumulation may occur through the enhanced permeability and retention effect, while active targeting ligands enhance tumor‐specific recognition and cellular uptake. Therefore, tumor‐targeting nanomedicine strategies have focused on functionalizing nanoparticles with specific ligands, antibodies, or small molecules that recognize overexpressed receptors on cancer cells [293, 294]. In parallel, stimuli‐responsive and externally actuated systems enable on‐demand drug release within pathological niches, further increasing intratumoral bioavailability and reducing off‐target toxicity [276]. Chen developed a HCC‐on‐a‐chip platform using a decellularized liver extracellular matrix scaffold, spatially organizing HepG2 tumor cells and L02 normal hepatocytes to evaluate magnetic field‐guided delivery of Fe_3_O_4_‐integrated liposomal nanomedicine. Confocal fluorescence imaging demonstrated efficient nanoparticle internalization within HepG2 cells while magnetic guidance enabled tumor‐selective nanoparticle enrichment. The predictive capability of this model for preclinical nanomedicine evaluation was further supported by its consistent therapeutic outcomes with cellular and animal studies [295].

### 5.4 Immunotherapy

Cancer cells evade immune surveillance through diverse mechanisms, including the secretion of immunosuppressive factors, upregulation of immune checkpoint molecules, and dynamic remodeling of the TME to foster tolerance. In response, multiple therapeutic strategies have been developed to reactivate the immune system and counteract immune evasion, such as immune checkpoint blockade, immunomodulatory drugs, cancer vaccines, adoptive cell transfer (ACT) therapy, and oncolytic virotherapy. While many of these approaches have demonstrated clinical efficacy in controlling or even reversing tumor progression, variable patient responses, significant adverse effects, and the development of therapeutic resistance still influence the quality of life of patients in the terminal stage [296, 297]. Consequently, there is an urgent need to develop advanced in vitro models that enable personalized immunotherapy, predict clinical outcomes, and facilitate the exploration of novel combination immunotherapies.

Immune checkpoint inhibitors (ICIs) disrupt inhibitory receptor–ligand interactions, thereby relieving immunosuppression and reinvigorating antitumor immunity, primarily through the restoration of T‐cell activation and cytotoxic function [297]. Therapies targeting the programmed cell death protein 1 (PD‐1) receptor and its ligand PD‐L1 have become a cornerstone of immunotherapy for multiple advanced malignancies [298]. Despite the substantial clinical success of ICIs, key uncertainties remain regarding who benefits most, under what conditions, and for how long. Although numerous studies have explored predictive biomarkers of therapeutic response, these cannot fully capture intratumoral heterogeneity, interpatient variability, or the dynamic evolution of the tumor immune microenvironment during treatment [299]. To address this limitation, Cui et al. engineered a microfluidics‐based 3D GBM‐on‐a‐chip model that integrated molecularly distinct patient‐derived GBM cells and tumor‐associated macrophages (TAMs) within a hyaluronan‐rich Matrigel matrix, together with perfusable brain microvessels and circulating IL‐2‐activated allogeneic CD8^+^ T cells. Under nivolumab‐based treatment, CD8^+^ T‐cell extravasation was quantified by time‐lapse imaging, whereas T‐cell activation was assessed by immunostaining. Therapeutic responses were further evaluated by profiling immunosuppressive mediators and measuring caspase‐3/7‐dependent GBM‐cell apoptosis. By integrating dynamic immune‐cell trafficking with molecular and functional readouts, this platform enabled a multidimensional and mechanism‐informed assessment of immunotherapy efficacy [300].

The fundamental objective of in vitro immunomodulatory drug screening is to recapitulate the interaction between primary tumor sites and the immune system. Sabhachandani et al. demonstrated differential responses of lymphoma to lenalidomide in the presence or absence of immune‐related cells using an alginate‐based microfluidic chip model [157]. Separately, Pece et al. established a 3D co‐culture system by seeding MSCs together with Reed‐Sternberg/Hodgkin lymphoma (HL) cells within collagen‐based scaffolds, followed by treatment with a combination of ADAM10 inhibitors and brentuximab‐vedotin. In this model, the reduction of key ADAM10 substrates was quantitatively assessed via ELISA of culture supernatants, which correlated with increased apoptosis of HL cells, indicating a synergistic combinatorial effect between ADAM10 inhibitors and the anti‐CD30 antibody [301]. Collectively, such co‐culture models offer a robust platform for the dynamic evaluation of both tumor cell behavior and the functional status as well as viability of immune components in response to immunotherapeutic interventions.

ACT therapy involves the ex vivo modification and expansion of autologous or allogeneic immune cells for subsequent reinfusion, aiming to achieve precise elimination of tumor cells. However, the efficacy of ACT strategies, especially against solid tumors, remains substantially limited, which is attributed to the dense ECM, highly immunosuppressive TME, and considerable tumor heterogeneity alongside antigen escape mechanisms [296]. These formidable challenges underscore the critical need for sophisticated preclinical models that accurately replicate these pathophysiological conditions, thereby advancing therapeutic development. Miller et al. utilized a 3D renal cell carcinoma on‐a‐chip model to recapitulate key challenges in adoptive T cell therapy for solid tumors. In this system, tumor spheroids were embedded within a tunable collagen ECM to mimic the desmoplastic tumor stroma. It was demonstrated that the infiltration capacity of engineered chimeric antigen receptor T cells was significantly impeded by dense collagen matrices (≥2.5 mg/mL), which simulate the increased tissue stiffness characteristic of solid tumors [302]. In contrast, 3D scaffold‐based culture systems offer a promising solution to overcome the ex vivo expansion bottlenecks in adoptive cell therapy. Notably, biodegradable HA‐based scaffolds have been demonstrated to significantly enhance the expansion fold, viability, and long‐term persistence of NK cells. The cytokine‐free system promotes the formation of multicellular clusters. It upregulates activation‐associated gene expression, thereby further augmenting the secretory profile of immunologically active molecules and the tumor‐lytic capacity of NK cells [302, 303, 304].

### 5.5 Precision Oncology

Despite major advances in genomics‐guided targeted therapies and immunotherapies, clinical benefit remains limited for many patients because of pronounced inter‐patient heterogeneity, uneven distributions of actionable mutations, and the frequent emergence of multidrug resistance in advanced disease [161]. These challenges have driven a shift toward patient‐specific management strategies based on individualized ex vivo models. Although patient‐derived xenografts have historically served this purpose, their long establishment times, clonal drift, and variable stability limit real‐time clinical utility [288, 292].

The integration of patient‐derived cancer cells with dECM scaffolds provides a relatively rapid and accessible platform for recapitulating clinically relevant treatment responses [46, 290, 305, 306, 307]. Shie et al. cultured the patient‐derived metastatic brain tumor cells on the decellularized adipose tissue and prospectively compared ex vivo drug responses with the corresponding clinical outcomes. The predicted responses showed an accuracy of 73.3%, with a sensitivity of 75.0% and a specificity of 71.4%, indicating its potential for individualized drug screening [305]. Nevertheless, the absence of immune components and angiogenic mechanisms restricted the assessment of immunotherapy, cell therapy, and anti‐angiogenic treatment, thereby limiting the platform's applicability across diverse therapeutic modalities.

Patient‐derived scaffolds (PDSs) are generated from patient tumor tissues through decellularization procedures, which preserve the architectural and biochemical features of the original lesion and support multicellular co‐culture. These platforms enable the assessment of how individualized TME modulates antitumor therapeutic responses [308, 309]. Leiva and his colleagues cultured the ER‐positive breast cancer cell line MCF7 within PDS obtained from 64 breast cancer patients and evaluated chemotherapy responses following doxorubicin treatment. Gene expression profiling demonstrated that surviving cells exhibited increased expression of CSC‐associated and EMT‐related markers. Associations between treatment‐induced molecular changes in PDS cultures and disease‐free survival were further identified. Nevertheless, the absence of immune and vascular compartments limits the capacity of PDS models to comprehensively recapitulate patient treatment responses [310]. Moreover, using patient‐derived matrices as bioinks for 3D bioprinting enables rapid and scalable fabrication of individualized tumor models and higher‐throughput assessment of patient‐specific therapeutic sensitivity [290]. To minimize the structural and functional disruption typically associated with conventional cell‐dissociation protocols, Choi et al. adopted a non‐dissociative mechanical mincing approach for patient‐derived tumor tissue, followed by immediate combination with gastric tissue‐derived dECM. Histological analyses confirmed that the bioprinted models retained the characteristic features of the original tumors. The platform enabled the evaluation of multiple single and combination therapies within two weeks, yielding drug‐response profiles consistent with clinical outcomes. Collectively, this approach provided a rapid, scalable, and reproducible preclinical platform for patient‐specific therapeutic assessment [307]. Another innovative approach is the unique tissue engineering technique termed cell‐assembled viscous tissue by sedimentation, developed by Takahashi et al. This method leverages the synergistic interaction between collagen and heparin to facilitate the rapid, one‐step assembly of cells into cohesive 3D tissue constructs [306].

### 5.6 Radiotherapy

Radiotherapy serves as a critical modality in cancer treatment, achieving precise elimination of tumor cells by inducing DNA damage through ionizing radiation, modulating the TME, and activating immune responses. Therefore, evaluating the radiosensitivity of tumor cells is essential for optimizing therapeutic decisions and improving patient prognosis. In recent years, a growing number of studies have focused on utilizing in vitro models to assess tumor cell responses to radiotherapy and predict clinical outcomes [311]. Conventional 2D cell culture systems possess inherent limitations in recapitulating the complex biology of tumors in vivo and often fail to reproduce key features of the TME. In contrast, 3D in vitro models have evolved from simple “cell containers” into “miniature tumor” systems capable of closely mimicking the organizational structure and function of real tumors. By reconstructing critical pathophysiological elements such as hypoxic gradients, cell–cell interactions, ECM properties, and tumor heterogeneity, these models offer a significantly superior platform for assessing and predicting radiosensitivity under conditions that better resemble the in vivo context.

3D models are capable of actively simulating and quantifying the decisive influence of key microenvironmental factors on radiosensitivity, thereby allowing in‐depth exploration of the mechanisms through which the microenvironment confers resistance. For instance, by adjusting the size of alginate hydrogels to create a distinct hypoxic environment, the hypoxic region demonstrates higher cell survival following radiation therapy, thereby mechanistically linking hypoxia to radioresistance [123]. Furthermore, by adjusting the composition of collagen/alginate composite hydrogels, the influence of physical properties such as ECM stiffness on radiation response can be systematically investigated. Studies have shown that nasopharyngeal carcinoma cells cultured within softer ECM substrates exhibit stronger radioresistance, accompanied by cell cycle arrest and upregulation of stem cell markers. This reveals ECM stiffness as a novel, composition‐independent mechanism contributing to treatment resistance [312]. Similarly, 3D models can also be employed to understand how specific molecular or genetic backgrounds influence radiosensitivity. For example, using 3D culture techniques to investigate the radiation sensitivity of HPV‐positive OSCC cells versus HPV‐negative OSCC cells, the study found that irradiated HPV‐positive cells exhibited a higher proportion of cells in the G1/S phase and demonstrated greater sensitivity to radiation compared with the negative cell lines [313].

Furthermore, cutting‐edge 3D platforms are striving to directly link in vitro assessments with clinical prognosis, advancing toward individualized prediction. By rapidly constructing miniature tumor models using patient‐derived cells on standardized 3D pillar/well arrays, a radiotherapy response index, RTauc, can be quantitatively calculated within approximately two weeks [314]. This index directly quantifies the survival capacity of tumor cells during simulated radiotherapy, enabling the rapid prediction of a patient's actual response to radiation before treatment. It successfully transforms the results of an in vitro functional test into a predictive biomarker that shows a statistically significant association with real post‐radiotherapy patient outcomes, such as recurrence and survival. However, the current validation of this model is largely based on patient cohorts who underwent surgery, and its predictive efficacy in patients receiving definitive radiotherapy still requires confirmation through prospective studies. Additionally, its representation of the TME remains relatively simplified and may not fully capture the complex mechanisms of radioresistance in vivo.

3D models therefore offer a radiobiological platform that surpasses 2D systems by incorporating microenvironmental modifiers of radiation response. To fully realize their clinical predictive potential, these models should incorporate patient‐derived cells, standardized radiation dosing protocols, and longitudinal tracking of survival and DNA repair. Linking in vitro radioresponse biomarkers with clinical imaging and outcome data will be crucial.

### 5.7 Drug Resistance

Drug resistance remains a central cause of cancer treatment failure. It is a complex process involving multiple mechanisms, among which those closely associated with the TME include EMT, dysregulated cellular energy metabolism, immune evasion, tumor‐promoting inflammation, and suppression of cell death [315]. Currently, most preclinical evaluations of anticancer drugs still rely primarily on conventional 2D cell culture systems, which offer limited predictive capacity [257]. Therefore, developing in vitro drug evaluation models that can predict individual sensitivity to anticancer agents is of critical importance. For example, researchers have developed a novel drug screening model based on 3D bioprinting, which enables the quantitative assessment of antitumor drug efficacy against drug‐resistant MCF‐7 BC spheroids formed within printed hydrogels [316]. In this context, 3D culture models have demonstrated remarkable advantages in mimicking clinical drug resistance [317]. Studies have shown that, compared to 2D models, cells cultured in 3D often exhibit significantly enhanced drug resistance, with half‐maximal inhibitory concentration (IC50) values that align more closely with clinically observed levels [46, 257, 318]. By reconstituting spatial heterogeneity, ECM interactions, stem cell niches, and complex cell–cell communication, 3D models not only enable more accurate prediction of clinical resistance phenotypes but, more importantly, provide an indispensable and physiologically relevant platform for systematically decoding the underlying mechanisms of drug resistance [257, 319].

The mechanisms underlying tumor drug resistance are complex and closely associated with the regulatory role of the ECM. Utilizing 3D models to deeply investigate the function of the ECM in the development of drug resistance can provide promising strategies for overcoming resistance. One study found that 3D collagen culture enhances the stemness and drug resistance of glioma cells, establishing it as a valuable model for tumor mechanism research and in vitro drug sensitivity testing [319]. In the bioprinted cancer models utilizing human adipose‐derived MSCs and BC cells, the supplementation of collagen I was found to facilitate the creation of more drug resistance‐relevant cancer models [320]. Furthermore, ECM stiffness also significantly influences drug resistance. For instance, Tianjiao Zeng et al. explored the effect of hydrogel stiffness on the chemoresistance of BC cells to doxorubicin, revealing that BC cells cultured in high‐stiffness hydrogels exhibited higher survival rates and elevated P‐glycoprotein mRNA expression compared to those in low‐stiffness hydrogels [321]. On the other hand, Sofia N. Luna et al. employed HA hydrogels to study how matrix rigidity induces dormancy and promotes resistance to paclitaxel or lapatinib in brain‐metastatic BC spheroids [322]. Together, these studies deepen our understanding of how matrix stiffness regulates drug‐resistant behaviors of cancer cells within 3D microenvironments, highlighting the critical role of ECM mechanical properties in modulating cell–matrix interactions and drug‐resistant phenotypes [108].

3D culture models based on biomimetic scaffolds, by reconstructing cell–cell and cell–ECM interactions, provide a powerful platform for systematically uncovering tumor drug resistance mechanisms within a microenvironment that more closely resembles in vivo conditions. First, 3D models are capable of identifying novel drug resistance signaling pathways that are difficult to detect in conventional 2D cultures. For example, in 3D “spiky” spheroids formed from colorectal cancer HCA‐7 cells, cetuximab resistance is not driven by known genetic mutations but rather by aberrant phosphorylation of the MET and RON receptor tyrosine kinases. This resistance can be effectively reversed by combining cetuximab with the dual MET/RON tyrosine kinase inhibitor crizotinib [323]. Second, 3D models are more adept at recapitulating drug‐resistant phenotypes associated with CSC properties and EMT. In BC 3D spheroid models, the novel compound LQB‐223 effectively inhibits cell migration, reduces viability, and downregulates multiple resistance‐related proteins, including XIAP, c‐IAP1, and Mcl‐1, while also modulating the expression of EMT‐related markers. This indicates that the compound can overcome resistance by interfering with cell plasticity [324]. Third, 3D models enable precise dissection of resistance mechanisms driven by specific biochemical components of the ECM. In biomimetic microgels incorporating decellularized hepatocellular carcinoma matrix (DHCM), DHCM upregulates the expression of aquaporin 3, which in turn activates the mTOR signaling‐associated autophagy pathway, thereby significantly enhancing the survival of HCC cells under chemotherapeutic stress [325]. Fourth, 3D models also reveal the fundamental impact of the mode of cell–ECM interaction itself on drug sensitivity. Comparing the response of colorectal cancer cells to vemurafenib in 2D versus 3D environments shows that the unique cell–ECM interaction pattern in 3D culture alters the drug‐induced enhancement of FAK tyrosine phosphorylation observed in 2D, which is a pro‐resistance signal, thereby affecting drug efficacy [326].

3D resistance models thus reveal that the ECM is not merely a physical scaffold but an active instructor of therapeutic failure. Moving forward, these systems should be used to evaluate ECM‐targeted adjuvant strategies that enhance treatment sensitivity. Additionally, coupling 3D drug testing with spatial transcriptomics and proteomics could uncover microenvironment‐driven resistance signatures predictive of clinical relapse.

### 5.8 Cancer Metastasis and Recurrence

Although advances in cancer treatment have significantly improved outcomes for patients with early‐stage lesions, the risk of recurrence and death due to relapse remains high [327]. Therefore, understanding the impact of various treatment modalities on cancer cells and the mechanisms underlying cancer recurrence is crucial. Some patients still experience recurrence after radiotherapy. Zhu et al. developed ECM hydrogels derived from dECM and observed changes in ECM components, including collagen I, IV, and VI, as well as fibronectin, following irradiation. Moreover, when cultured with macrophages in dECM hydrogels, M2 macrophage polarization was induced, accompanied by further proliferation. This study elucidates the mechanism by which ECM alterations after radiotherapy contribute to BC recurrence [328]. Additionally, chemotherapy can promote cancer cell survival and dissemination by inducing tumor‐intrinsic and extrinsic changes. Fatherree et al. constructed dECM from chemotherapy‐treated mice to investigate how cytotoxic chemotherapies, particularly paclitaxel and doxorubicin, drive recurrence and metastasis in TNBC by altering tumor ECM composition. They proposed a novel mechanism of “chemotherapy‐induced metastasis”: while attacking cancer cells, cytotoxic chemotherapies remodel the tumor ECM, leading to upregulation of proteins such as collagen IV. The altered ECM, through activation of the integrin‐Src/FAK signaling axis, endows residual cancer cells with enhanced motility and invasive capabilities, potentially paving the way for local recurrence or distant metastasis after treatment [329].

These studies underscore that therapy‐induced ECM remodeling can inadvertently create a pro‐metastatic niche. To mitigate this risk, 3D models that capture post‐treatment matrix evolution and its impact on residual cells are urgently needed. Such platforms could enable the screening of adjuvant therapies designed to normalize the ECM and prevent recurrence following standard interventions.

## 6 The Framework of Designing Scaffold‐Based Models

A practical framework is needed to guide the rational design of scaffold‐based 3D tumor models according to specific biological applications. Therefore, this section proposes a comparative framework that links the intended research question with key design considerations, recommended materials, cell composition, and representative models (Table 4).

**TABLE 4:** A comparative framework for the design of scaffold‐based 3D tumor models according to different biological applications.

Biological application	Design considerations	Recommended materials	Cell composition	Representative models
Tumor invasion and metastasis	ECM stiffness Adhesion ligands Migration paths Endothelial barrier Vascularization	Tissue‐mimetic hydrogels Engineered scaffold systems dECM	Cancer cell lines Stromal cells Endothelial cells Fibroblasts Circulating tumor cells	PCL scaffold‐based CTC culture model [50] Bone‐on‐a‐chip metastatic niche model [254] Lung cancer intravasation‐on‐a‐chip model [248]
Drug response evaluation	Tumor–stroma interactions Multicellular co‐culture Drug penetration Vascularization	Tissue‐mimetic hydrogels Engineered scaffold systems dECM	Cancer cell lines Stromal cells Endothelial cells Fibroblasts Normal tissue cells Cancer stem cells	Decellularized hepatocellular carcinoma‐on‐a‐chip model [295] Uterine sarcoma 3D spheroid model [284]
Immunotherapy evaluation	Immune‐cell incorporation Multicellular co‐culture Immune‐cell trafficking Immune‐cell persistence	Tissue‐mimetic hydrogels Engineered scaffold systems dECM	Cancer cell lines CD8+ T cells Monocytes NK cells TAMs Stromal cells Endothelial cells	GBM‐on‐a‐chip model [300] Carcinoma‐on‐a‐chip model [302] 3D engineered HA‐based niche for cell expansion model (3D‐ENHANCE) [304]
Patient‐specific therapeutic response	Tumor heterogeneity Clinical correlation	Patient‐derived decellularized tumor scaffolds Tissue‐specific dECM	Patient‐derived tumor cells Cancer cell lines Endothelial cells Stromal cells	Breast cancer patient‐derived scaffold model [309] Colorectal cancer patient‐derived scaffold model [308]
High‐throughput drug screening	Reproducibility Standardization Automation Platform compatibility	dECM‐based bioinks Preformed micro‐scaffolds ECM‐mimetic hydrogels	Cancer cell lines Stromal cells	3D microtumor array model [330] Leukemia droplet microfluidic model [276] Bioprinted breast cancer model [273]

Beyond model selection, a major challenge in designing scaffold‐based 3D tumor models is determining whether observed phenotypes reflect intrinsic tumor biology or effects induced by scaffold materials. As discussed in Section 3.5, scaffold composition, mechanical properties, and biochemical cues can influence cellular states and therapeutic responses. One practical approach is to compare cellular responses across different culture platforms to assess whether key phenotypes remain consistent across distinct material environments. Svanström et al. compared the responses of MCF7 breast cancer cells cultured in PDSs, 3D‐printed scaffolds, and Matrigel under normoxic and hypoxic conditions. The different platforms produced distinct molecular response patterns related to metabolism, proliferation, cancer stemness, and hypoxia. qPCR and Western blot analyses further showed that cells cultured in 3D‐printed scaffolds exhibited molecular profiles more similar to those of PDS‐cultured cells, whereas Matrigel produced more distinct responses, particularly under hypoxic conditions. Such comparisons can reveal material‐dependent deviations in cellular responses [331]. A more rigorous validation strategy involves direct benchmarking of scaffold‐based models against matched tumor tissues. In a patient‐derived pediatric brain tumor model based on 3D silk fibroin scaffolds, transcriptomic profiles were directly compared with the matched primary tumor tissues. RNA‐seq showed that selected medulloblastoma models closely reproduced the gene expression profiles of their corresponding tumors, with less than 1% of genes differentially expressed. These findings support the application of omics‐based comparison with patient‐derived tissues as a complementary strategy for validating scaffold‐based models [332].

## 7 Current Challenges and Future Perspectives

### 7.1 Persisting Challenges

Despite substantial advances, scaffold‐based 3D in vitro models continue to face major translational barriers before they can function as reliable, decision‐grade tools in preclinical research. A central limitation is the absence of standardized and benchmarked fabrication workflows. Current practices vary significantly across different cancer types, biomaterials, and manufacturing techniques, which undermines reproducibility and limits clinical translatability. For most cancer subtypes, indication‐specific standard operating procedures are lacking, and there is no consensus on the optimal biomaterial classes, formulations, or architectural parameters required to recapitulate native tumor niches. Moreover, critical sources of variability remain poorly controlled, while quantitative success criteria for model validation, such as physiologically relevant vascular permeability, oxygen gradients, or matrix signatures, are rarely predefined. The absence of validated outcome surrogates that are prospectively linked to clinical endpoints, combined with limited inter‐laboratory harmonization through ring trials or standardized reporting, further constrains the predictive utility and iterative development of these platforms.

Furthermore, existing scaffold models offer only an incomplete recapitulation of the complex TME. They typically fail to replicate the biochemical specificity, anisotropy, dynamic remodeling, and multicomponent architecture of the ECM at the same time, limiting their ability to faithfully model cell migration, mechanotransduction, and drug penetration. Vascularization strategies, though advancing, frequently lack physiologic barrier functions, lymphatic networks, and relevant shear stresses, thereby inadequately representing transport and metastatic processes. The full cellular diversity and interactive dynamics of the TME, including patient‐matched stromal and immune cell subsets, stable co‐culture systems, and critical feedback loops, remain challenging to incorporate and sustain in vitro. Additionally, most platforms do not account for essential systemic and evolutionary contexts, such as pharmacokinetic–pharmacodynamic relationships, multi‐organ crosstalk, and long‐term clonal selection, which are crucial for modeling therapeutic response and resistance over time.

Scalability represents a further barrier to the broader adoption of scaffold‐based 3D tumor models. Increasingly faithful recapitulation of the native TME requires specialized equipment, sophisticated biomaterials, and extended culture periods, limiting cost‐effective and scalable production. This challenge is particularly pronounced for patient‐derived systems, where limited specimen availability, variable cell viability, and restricted ex vivo expansion constrain experimental throughput and replication. 3D bioprinting provides an important technological basis for high‐throughput platform development by enabling the rapid, reproducible, and spatially controlled fabrication of multiple tissue constructs. However, its scalable implementation requires standardized bioink formulations and printing protocols to ensure consistent model quality. Moreover, many existing platforms remain poorly compatible with standardized multiwell formats, automated liquid handling, and high‐content imaging, thereby limiting their integration into large‐scale drug‐screening workflows.

Batch differences in scaffold‐based models can alter their mechanical and biochemical properties, introducing uncertainty into cell behavior and drug‐response measurements. For naturally derived hydrogels, poorly defined compositions can produce marked variation in molecular composition and local stiffness at both inter‐ and intra‐batch levels [333]. Synthetic hydrogels offer a means of minimizing such material‐driven variation through controlled fabrication and more uniform scaffold performance across batches. Despite these improvements, non‐standardized scaffold preparation and culture procedures can introduce process‐related variability. In particular, differences in seeding concentration, seeding technique, and culture duration can alter cellular organization and morphology, thereby compromising experimental repeatability and cross‐laboratory reproducibility [334]. Furthermore, the complexity of in vitro scaffold‐based models can introduce additional variability through differences in medium replenishment, oxygen availability, pH, and soluble signaling gradients. Automated bioprinting systems are promising solutions to improve reproducibility; however, the bioink rheological properties, temperature fluctuations, and nozzle stability may affect deposition accuracy and construct fidelity. Collectively, these challenges underscore the need for standardized model evaluation, real‐time process monitoring, and predefined batch‐release criteria to enable the timely identification of nonconforming batches.

Conventional scaffold‐based models can restrict therapeutic transport due to dense ECM‐like networks, limited matrix permeability, and extended transport distances within the constructs. In simple scaffold‐based 3D models, therapeutic agents primarily rely on passive diffusion from the surrounding medium, resulting in heterogeneous drug distribution. Vascularized platforms enable perfusion‐mediated delivery throughout the construct, achieved through EC co‐culture, bioprinting‐based vascular fabrication, and microfluidic perfusion systems [335]. However, current vascularization strategies remain insufficient to fully reproduce tumor‐specific vascular features and dynamic vessel remodeling [336]. Consequently, these limitations compromise the accuracy of therapeutic response evaluation, leading to unreliable predictions of drug efficacy.

The physicochemical properties of scaffold materials could affect the reliability of pharmacological assessments by modifying molecular transport and cellular exposure. Scaffold components interact with therapeutic molecules through adsorption or retention effects [337]. For example, PDMS, a commonly used material in microfluidic platforms, has been shown to adsorb and retain small hydrophobic molecules during prolonged culture, thereby altering drug gradients and dose–response profiles [148]. Moreover, the release of PDMS‐derived components into the culture environment may further influence cellular behaviors, including proliferation, adhesion, and differentiation. Alternative substrates, such as cyclic olefin copolymer and polymethyl methacrylate, together with PDMS‐free hydrogel‐based fluidic architectures, have been explored to reduce material‐mediated interference while preserving controlled molecular delivery [125, 338]. Taken together, material selection represents a critical design consideration in scaffold‐based 3D model construction, with direct implications for model reproducibility and translational relevance.

### 7.2 Future Directions

#### 7.2.1 Advanced Scaffold Systems: 4D‐Bioprinting, “Smart” Responsive Materials (to pH, Enzymes), and Dynamic Culture Platforms

4D printing refers to an advanced additive manufacturing technology that enables the creation of constructs with programmable, time‐dependent transformations in shape, structure, or function. By incorporating the dimension of time, it allows printed objects to undergo programmable and predictable transformations in shape, structure, or function over time when exposed to external stimuli such as temperature, humidity, light, pH, or electric fields [339, 340, 341, 342, 343, 344]. 4D‐printed materials hold significant value in tumor research, as they enable the construction of biomimetic structures that can dynamically respond to environmental changes. This enables more precise and dynamic simulation of native tissues in vitro, which plays a critical role in investigating tumor progression mechanisms and facilitating drug screening. Zhao et al. developed a 4D cell culture system to mimic the mechanical stress of gastric peristalsis in vitro. They engineered a magnetically responsive hydrogel by incorporating magnetic nanoparticles into an RGD‐functionalized alginate matrix. When exposed to a rotating magnetic field, this scaffold applied dynamic stress to cultured gastric cancer spheroids. Compared to conventional 3D cultures, the 4D model significantly altered cell growth kinetics by modulating PCNA and p53 expression, and enhanced the stem‐like properties of cancer spheroids, demonstrating its utility in modeling the biomechanically active TME [345]. By combining rational design of innovative materials with patient‐derived grafts, researchers used 4D printing technology to optimize in vitro cell culture processes, explore personalized treatment strategies, and promote the translation of preclinical models [346]. Furthermore, considering the interactions between tumors and their microenvironment, one of the key objectives of 4D printing is to engineer in vitro culture models that can dynamically respond to stimuli arising from tumor cell metabolism or interactions with surrounding cells.

#### 7.2.2 Multi‐Omics Integration: Coupling 3D Models With Single‐Cell RNA Sequencing and Spatial Transcriptomics for Deep Mechanistic Insight

Multi‐omics data have profoundly advanced tumor research by enabling a comprehensive, multilayered characterization of cancer biology. The integrative analysis elucidates the complex mechanisms underlying tumor heterogeneity, evolution, microenvironment remodeling, immune evasion, and therapy resistance [347, 348]. In the construction and validation of 3D models, multi‐omics analyses systematically uncover molecular characteristics across genomic, transcriptomic, proteomic, and metabolomic levels, thereby affirming the high biological fidelity of these models in recapitulating the TME [349, 350, 351]. Moreover, integrating bioinformatics with 3D modeling offers critical insights into tumor cell–ECM interactions, the architecture of cell–cell communication networks, and the mechanistic basis underlying phenotypic alterations in cancer cells [352, 353, 354, 355]. Yuan and colleagues employed 3D bioprinting to spatially organize BC cells and tumor‐associated stromal cells, thereby constructing a vascularized BC model. Spatial transcriptomic analysis of this model decoded the molecular basis of its spatial heterogeneity. Notably, cancer cells within the stromal region, compared to those in cancer cell‐rich regions, upregulated the oxidative phosphorylation pathway and exhibited enhanced resistance to the chemotherapeutic agent doxorubicin [356]. The deep integration of multi‐omics technologies with 3D in vitro models will enable the systematic dissection of multidimensional dynamics, including spanning transcriptional, proteomic, metabolomic, and genetic layers, within a unified model system. Concurrently, by accumulating multi‐omics reference datasets across engineered 3D culture systems, a quantitative evaluation framework based on molecular signatures can be established, thereby advancing the standardization of model assessment and validation.

#### 7.2.3 Toward a “Clinical Trial in a Dish”

Large libraries of scaffold‐based patient models could be used to stratify patients and optimize trial design. These models indicate preclinical oncology by enabling individualized assessment of therapeutic responses before patient enrollment. This momentum coincides with a regulatory shift toward embracing non‐animal New Approach Methodologies in preclinical assessment. Notably, the U.S. FDA Modernization Act 2.0 explicitly permits alternatives to animal testing for drug applications and has spurred FDA initiatives to advance in vitro and computational approaches for evaluating safety and efficacy.

A major challenge in traditional oncology trials is patient heterogeneity, which often dilutes efficacy signals from responsive subgroups during full‐cohort analysis, leading to trial failure despite genuine activity in specific strata. To address this, we propose a next‐generation strategy centered on establishing large, curated libraries of biomimetic, scaffold‐based patient models. These libraries serve as a prospective platform for rational clinical trial design and patient stratification.

Under this framework, primary human tumors representing diverse clinical and molecular phenotypes would be procured under standardized protocols and engineered into physiologically relevant constructs. Techniques such as dECM scaffolds, microfluidic vascularization, and 3D bioprinting would be employed to preserve native tissue architecture and enable stable multicellular co‐culture. Each patient‐specific model would be annotated with comprehensive clinical metadata and linked to longitudinal clinical outcomes. High‐content assay data, encompassing responses to single agents and drug combinations, dose‐schedule sensitivity, pharmacodynamic biomarkers, and emergent resistance patterns, would be integrated into a relational model‐to‐clinic database.

This integrated resource would enable a priori optimization of trial design. By querying the database, researchers could: (i) define molecular or phenotypic inclusion criteria to enrich for predicted responders; (ii) identify optimal dosing regimens and drug combinations that maximize therapeutic efficacy within defined subgroups; and (iii) determine empirically guided timepoints for pharmacodynamic sampling and interim analyses. Potential treatment regimens could be screened across the entire model library before patient enrollment, enabling high‐throughput identification of sensitive patient subsets and yielding more accurate estimates of expected effect sizes.

However, the establishment of large‐scale scaffold‐based patient model libraries alone will not be sufficient to support clinical implementation. A critical prerequisite is prospective validation of the concordance between model responses and matched patient data, including treatment efficacy, resistance development, and survival‐related endpoints. To date, most clinical translation efforts involving scaffold‐based patient models have primarily focused on feasibility demonstration and retrospective validation [357, 358]. The discrepancies between model predictions and clinical responses have also been observed in some cases, partially attributable to incomplete recapitulation of the native tumor ecosystem. Furthermore, challenges including prolonged model generation timelines and the absence of standardized manufacturing and quality‐control frameworks remain major barriers to the routine clinical implementation of these platforms. Future prospective, multicenter, and large‐scale clinical trials will be required to establish whether scaffold‐based models can reliably guide treatment selection and ultimately improve patient outcomes.

## 8 Conclusions

Biomimetic scaffold‐based 3D models have become valuable platforms in cancer research by addressing several limitations of conventional 2D cultures and providing more physiologically relevant representations of the physical, biochemical, and cellular features of the TME. This review provides a structured overview of diverse biomaterial platforms, including natural and synthetic polymers, hybrid composites, and dECMs, together with design strategies for modeling key microenvironmental features such as matrix stiffness, hypoxia, multicellular crosstalk, angiogenesis, cancer stemness, and metastatic processes. These models have been increasingly applied to drug screening, mechanistic studies of therapeutic resistance, and the evaluation of personalized treatment strategies, immunotherapy, and radiotherapy. By bridging the gap between reductionist in vitro systems and the complexity of in vivo systems, they provide complementary information that is difficult to obtain from conventional reductionist models.

Despite these advances, critical challenges persist, including the lack of standardized fabrication workflows, incomplete recapitulation of the TME's dynamic and multicomponent nature, and scalability limitations for high‐throughput applications. Importantly, enhanced biological fidelity alone is insufficient to establish the clinical predictive validity of these models. Although several patient‐derived scaffold models have shown associations between ex vivo drug responses and clinical outcomes, their predictive value remains insufficiently validated in prospective clinical studies. Future integration of 4D bioprinting, smart responsive materials, and multi‐omics approaches will drive the next generation of these models, enabling dynamic spatiotemporal modulation of the TME and deep mechanistic insights into tumor heterogeneity and signaling networks. Large libraries of patient‐derived scaffold models may also provide an experimental framework for exploring patient stratification and trial optimization, while their clinical utility will require prospective validation. As interdisciplinary innovations continue to refine these systems, scaffold‐based 3D tumor models could serve as increasingly informative complementary platforms for translational cancer research and therapeutic development.

## Author Contributions

H.Z., X.L., C.H., W.W., and Q.C. conceived of and designed the study. H.T.Z. and Y.H. designed the figures and wrote the manuscript. H.T.Z., Y.H., J.L., P.L., X.L., H.Z., Z.X., L.Q., W.W., and Q.C. revised the manuscript. H.Z., C.H., and W.W. provided the funding support. All authors read, edited, and approved the final manuscript.

## Funding

This work was supported by the National Natural Science Foundation of China (NO. 82303610, 82503442), Chongqing Medical Youth Top Talent Program (NO. YXQN202558), Chongqing Youth Innovation Talent Project (NO. CSTB2025YITP‐QCRCX0096), Natural Science Foundation of Chongqing (NO. CSTB2025NSCQ‐GPX1144), Joint Funds for the Innovation of Science and Technology, Fujian Province (NO. 2023Y9299), and Natural Science Foundation of Fujian Province (NO. 2026J001210).

## Ethics Statement

The authors have nothing to report.

## Consent

The authors have nothing to report.

## Conflicts of Interest

The authors declare no conflicts of interest.

## Data Availability

Data sharing not applicable to this article as no datasets were generated or analysed during the current study.
